# Intratumoral and peritumoral radiomics for the pretreatment prediction of response to neoadjuvant chemotherapy in rhabdomyosarcoma: a multicenter retrospective cohort study

**DOI:** 10.1186/s13244-025-02178-0

**Published:** 2026-01-05

**Authors:** Ge Zhang, Yun Peng, Yan Su, Lin Mei, Jugao Fang, Yuanhu Liu, Huanming Wang, Hongcheng Song, Dong Guo, Guoxia Yu, Shengcai Wang, Xin Ni

**Affiliations:** 1 Department of Otolaryngology, Head and Neck Surgery, Beijing Children’s Hospital, Capital Medical University, National Center for Children’s Health, Beijing, China; 2 Department of Radiology, Beijing Children’s Hospital, Capital Medical University, National Center for Children’s Health, Beijing, China; 3 Department of Hematology Oncology Center, Beijing Children’s Hospital, Capital Medical University, National Center for Children’s Health, Beijing, China; 4 Department of Otolaryngology-Head and Neck Surgery, Beijing Tongren Hospital, Capital Medical University, Key Laboratory of Otolaryngology-Head and Neck Surgery (Capital Medical University), Ministry of Education, Beijing, China; 5 Department of Otolaryngology, Head and Neck Surgery, Shunyi Maternal and Children’s Hospital of Beijing Children’s Hospital, Beijing, China; 6 Department of Surgical Oncology, Beijing Children’s Hospital, Capital Medical University, National Center for Children’s Health, Beijing, China; 7 Department of Urological Surgery, Beijing Children’s Hospital, Capital Medical University, National Center for Children’s Health, Beijing, China; 8 Department of Orthopedics, Beijing Children’s Hospital, Capital Medical University, National Center for Children’s Health, Beijing, China; 9 Department of Stomatology, Beijing Children’s Hospital, Capital Medical University, National Center for Children’s Health, Beijing, China; 10 Laboratory for Pediatric Head and Neck Tumors, Capital Medical University, Beijing, China

**Keywords:** Pediatric rhabdomyosarcoma, MRI radiomics, Peritumoral, Neoadjuvant chemotherapy response prediction

## Abstract

**Background:**

Pediatric rhabdomyosarcoma (RMS), the most common soft-tissue sarcoma in children, exhibits heterogeneous responses to neoadjuvant chemotherapy (NAC), necessitating reliable biomarkers for early prediction. This multicenter study evaluates MRI-derived radiomic features of intratumoral and peritumoral regions to predict NAC response in the largest pediatric RMS cohort to date.

**Materials and methods:**

A retrospective analysis included 519 RMS patients from three Chinese centers. Radiologists manually segmented tumors and 2-mm peritumoral regions on standardized T1-weighted contrast-enhanced (T1CE) and T2-weighted fat-saturated (T2Fs) MRI sequences. PyRadiomics extracted 1015 radiomic features, with robustness ensured (ICC ≥ 0.80) and predictive features selected via LASSO regression. Twelve XGBoost models (intra-/peritumoral, multisequence) were developed, validated internally/externally, and compared using DeLong’s test, net reclassification improvement (NRI), and integrated discrimination improvement (IDI). SHAP analysis interpreted feature contributions. Clinical variables (age, fusion gene) were assessed for incremental value.

**Results:**

The T1CE-based combined intratumoral–peritumoral model (T1CE_IntraPeri2mm) demonstrated the best generalizability, achieving AUCs of 0.917 (training), 0.760 (internal validation), 0.837 (external test1) and 0.843 (external test2). It significantly outperformed intratumoral-only and multisequence fusion models in DeLong, NRI, and IDI analyses (all *p* < 0.05). The combined clinical-radiomic model did not provide incremental benefit (AUC: 0.843 vs. 0.838, *p* = 0.891). SHAP analysis indicated that features reflecting peritumoral structural irregularity and enhancement heterogeneity were key predictors of NAC resistance.

**Conclusion:**

T1CE-based peritumoral radiomics robustly predicts NAC response in pediatric RMS, emphasizing tumor-microenvironment interactions. This approach offers a non-invasive tool for personalized therapy stratification.

**Critical relevance statement:**

This study establishes peritumoral MRI radiomics as a critical predictor of chemotherapy response in pediatric rhabdomyosarcoma, addressing the unmet need for non-invasive biomarkers and advancing precision oncology through tumor-microenvironment interaction analysis in clinical radiology practice.

**Key Points:**

- Integrated tumor/peritumoral MRI features enhance neoadjuvant chemotherapy (NAC) response prediction.
- T1CE MRI best captures tumor-microenvironment treatment interactions.
- Non-invasive radiomics model outperforms clinical factors for therapy adjustment.

**Graphical Abstract:**

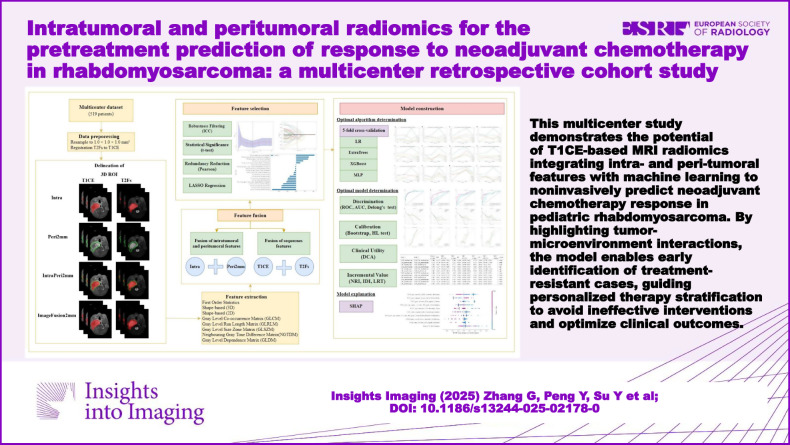

## Introduction

Rhabdomyosarcoma (RMS) is the most common soft-tissue sarcoma in children and adolescents, accounting for approximately 3–4% of all pediatric malignancies [1, 2]. Current RMS treatment strategies involve chemotherapy as the primary approach for cytoreduction and eradication of both macroscopic and microscopic metastatic disease, supplemented by surgery (where feasible) and radiation therapy (RT) to address microscopic local residual disease [3]. Given the difficulty of achieving complete resection at diagnosis for most RMS patients, neoadjuvant chemotherapy (NAC) is typically administered following pathological confirmation, with subsequent treatments tailored to individual responses. NAC offers potential advantages by reducing surgical morbidity and improving clinical outcomes; however, not all RMS cases respond favorably [4]. The ability to predict neoadjuvant treatment efficacy remains challenging, yet early identification of non-responders is crucial to avoid unnecessary and potentially harmful interventions and to optimize resources for patients who may benefit from NAC [5, 6]. Therefore, the development of reliable methods to evaluate the predictive value of imaging in assessing tumor response to neoadjuvant therapies is timely and relevant. Such advancements could transform RMS management, facilitating more effective and personalized treatment plans for each patient.

RMS tumors often exhibit heterogeneity on magnetic resonance imaging (MRI), with varying intratumoral patterns that are difficult to discern and articulate through traditional human observation [7]. Radiomics, an emerging field in imaging research, offers a solution to these challenges by providing tools for extensive, objective, and unbiased quantification of the STS radiophenotype across different imaging modalities [8]. Since its emergence in 2010 [9] and increased application in sarcoma research by 2015 [10], radiomics has shown promise in building predictive models to differentiate benign from malignant soft-tissue tumors, predict histologic grades (a critical prognostic factor in STS), assess responses to NAC or radiotherapy, and estimate survival rates and risks of distant metastasis [8, 11]. Notably, evidence from other cancers demonstrates significant associations between peri-lesional texture features and therapeutic resistance/poor prognosis, suggesting their potential as imaging biomarkers [12, 13]. However, the translational value of peritumoral radiomics remains underexplored in soft-tissue sarcomas—despite RMS ranking as the third most prevalent extracranial solid tumor in children, radiomic investigations specific to this entity, encompassing both intratumoral heterogeneity decoding and peritumoral microenvironment characterization, remain unexplored. To date, artificial intelligence applications in RMS have primarily focused on histopathological analysis [14–16]. For instance, Banerjee et al attempted to differentiate embryonal RMS (ERMS) and alveolar RMS (ARMS) subtypes using a deep learning-based computer-aided diagnosis (CADx) approach on multiparametric MRI images, but their study included only 21 pediatric patients [17].

Given the limited research in this area and the urgent need for effective tools to predict RMS responses to NAC, we conducted a multicenter retrospective study of 519 RMS patients—the largest radiomics cohort to date for this malignancy. Based on this cohort, we aimed to identify optimal imaging biomarkers for predicting NAC response, ultimately guiding personalized therapeutic strategies in pediatric RMS.

## Materials and methods

### Patients’ information

**Fig. 1:**
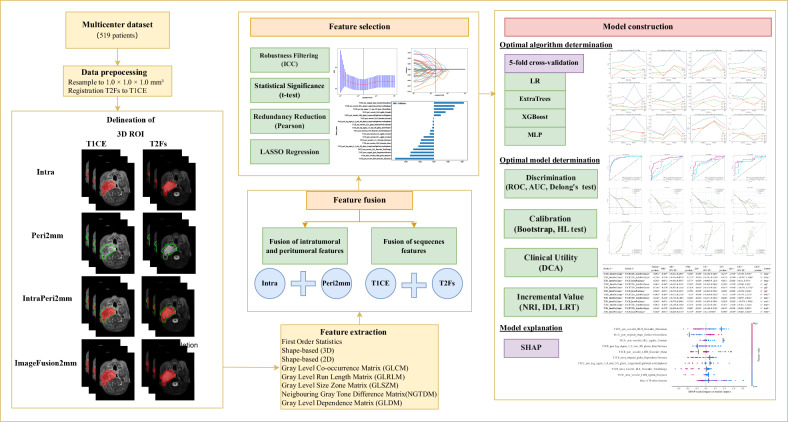
Workflow of the study. ROI, region of interest; T1CE, T1-weighted contrast-enhanced; T2Fs, T2-weighted fat-saturated; Intra, intratumoral regions; Peri, peritumoral regions; ICC, intraclass correlation coefficient; LR, logistic regression; XGBoost, extreme gradient boosting; MLP, multi-layer perceptron; ROC, receiver operator characteristic curve; AUC, area under the receiver operator characteristic curve; HL test, Hosmer–Lemeshow goodness-of-fit test; DCA, decision curve analysis; NRI, net reclassification improvement; IDI, integrated discrimination improvement; LRT, likelihood ratio test; SHAP, SHapley Additive exPlanations

**Fig. 2:**
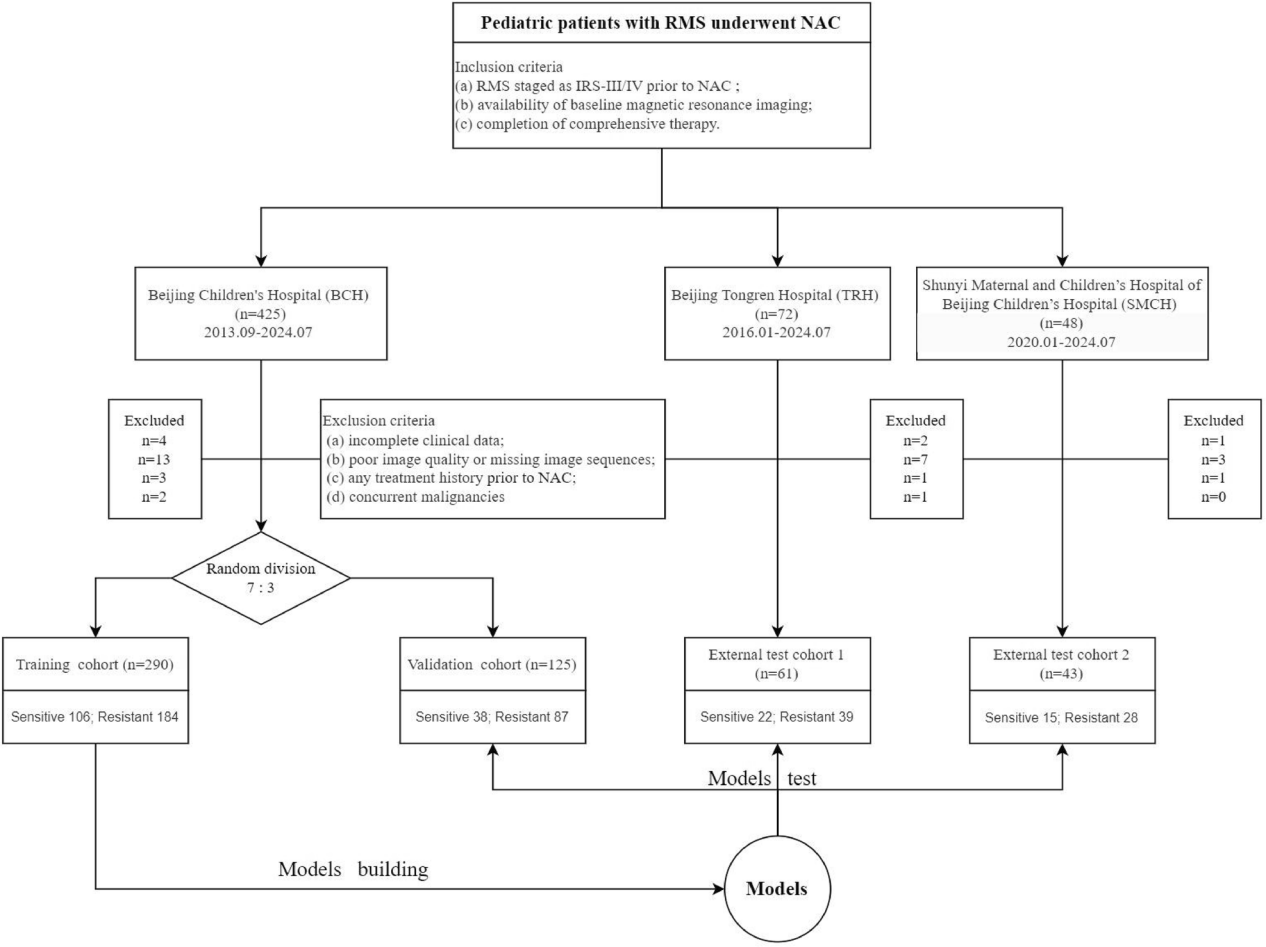
Flowchart of patient recruitment. RMS, rhabdomyosarcoma; IRS, Intergroup Rhabdomyosarcoma Study postsurgical grouping system; NAC, neoadjuvant chemotherapy

The study design is illustrated in Fig. 1. This retrospective study encompassed three cohorts from three academic medical centers in China. Patients with RMS treated at Beijing Children’s Hospital (BCH) between September 2013 and June 2024 were enrolled and randomly divided into a training set (70%, *n* = 290) and an internal validation set (30%, *n* = 125). An external test cohort 1 (*n* = 61) was collected at Beijing Tongren Hospital (TRH) from January 2016 to June 2024. An external test cohort 2 (*n* = 43) was completed by Shunyi Maternal and Children’s Hospital of Beijing Children’s Hospital (SMCH) from January 2020 to June 2024. The flowchart of patient selection is shown in Fig. 2. This retrospective study was approved by the institutional review boards of the respective institutions (BCH: [2022]-E-097-Y; TRH: TREC2024-KY093; SMCH: 2024-020-1), and the requirement for informed consent was waived. The details regarding sample size calculation, as well as the evaluation criteria for patient inclusion and response among enrolled patients, are provided in the Supplementary Methods.

### Image acquisition, pre-processing and segmentation

The patients included in this study underwent MRI scans using a 3.0-T MRI scanner (Discovery MR 750, General Electric) at BCH, a 3.0-T MRI scanner (Magnetom Skyra, Siemens Healthineers) at TRH, and a 3.0-T MRI scanner (Signa HDxt, General Electric) at SMCH. Due to the inherent limitations of retrospective studies, the types of MRI sequences collected varied depending on the anatomical region. Therefore, we selected T1CE and T2Fs, which were available for all patients. Detailed scanning parameters for each acquisition sequence are provided in Supplementary Table S1. To mitigate image quality differences arising from different scanning equipment and protocols, we utilized the Resample function in the SimpleITK library to resample the MRI images, unifying their voxel sizes to 1.0 × 1.0 × 1.0 mm³, and employed cubic B-spline interpolation (i.e., sitk.sitkBSpline) to ensure the quality of the resampled images. Tumor segmentation was performed manually by radiologists. A 2-mm peritumoral region was automatically generated from the tumor boundary and manually refined to exclude normal tissues. Detailed methods are in the Supplementary Methods.

### Radiomics procedure

#### Feature extraction and selection

In this study, quantitative imaging features were separately extracted from the intratumoral (Intra), peritumoral 2-mm margin (Peri2mm), and fused intratumoral–peritumoral 2-mm margin (ImageFusion2mm) regions on T1CE and T2FS MRI sequences. A total of 1015 radiomic features, including shape, statistical, and texture features, were extracted from each ROI using PyRadiomics (v3.1.0). Feature concatenation was employed to integrate intra-, peri-, and multisequence features into high-dimensional vectors. Four feature sets were constructed per sequence, and four cross-sequence feature groups were generated through integration, resulting in 12 feature groups in total. Feature selection was performed based on test-retest/inter-rater ICC (≥ 0.80), z-score normalization, significance testing (*p* < 0.05), collinearity reduction (r ≥ 0.90), and Lasso regression with cross-validation. A detailed feature extraction and selection methodology is provided in Supplementary Methods. The finally selected features from the 12 feature groups are presented in Supplementary Table S2 and Supplementary Fig. S1.

### Model building

Initially, we conducted a 5-fold cross-validation on the 12 screened feature groups to assess the stability of multiple classifiers across different data splits. Four classifiers with consistent performance were selected: Logistic Regression (LR), ExtraTrees, Extreme Gradient Boosting (XGBoost), and Multi-Layer Perceptron (MLP). Subsequently, we further compared the area under the receiver operator characteristic curve (AUC) performance of these four classifiers on the training, validation, and test sets to evaluate their generalization abilities (Supplementary Fig. S2). Ultimately, XGBoost demonstrated the best performance on both the validation and test sets and was selected as the final machine learning algorithm to construct 12 radiomics models. Clinical features were numerically mapped and analyzed using machine learning algorithms similar to those in the radiomics model. To construct the clinical model, univariate Logistic regression was initially employed, with significant factors (*p* < 0.1) being subsequently input into multivariate analysis. Ultimately, two features were selected: fusion_gene and pathology (Supplementary Table S3). Based on clinical correlations, we further incorporated variables explicitly associated with RMS prognosis, namely age, size, site, T, N, M, and IRS. Considering the established link between age and RMS from previous literature, age was categorized as a categorical variable for inclusion. However, due to the collinearity between IRS-III/IV (included in this study) and M, the final clinical variables incorporated were age, pathology, fusion_gene, size, site, T, N, and M. Among various algorithms, XGBoost demonstrated the best performance (Supplementary Fig. S3).

### Statistical analysis

Continuous variables were assessed for normality using the Shapiro–Wilk test, with independent *t*-tests applied to normally distributed data and Mann–Whitney U tests to non-normal data. Categorical variables were analyzed via chi-square or Fisher’s exact tests. Predictive performance was evaluated through Receiver Operating Characteristic (ROC) curves, with AUC differences between models tested by DeLong’s method. Bootstrapping (1000 iterations) generated 95% confidence intervals (CIs) for all metrics. Net Reclassification Improvement (NRI) and Integrated Discrimination Improvement (IDI) quantified incremental value over clinical benchmarks, while a likelihood ratio test (LRT) assessed the significance of adding radiomic features. Model calibration was validated using bootstrap-derived calibration curves and the Hosmer-Lemeshow goodness-of-fit test. Clinical utility was examined via Decision Curve Analysis (DCA) across probability thresholds (10–90%). Feature contributions and nonlinear interactions were interpreted using SHapley Additive exPlanations (SHAP). Analyses were conducted in Python 3.7.12 with scikit-learn 1.0.2 (machine learning), statsmodels 0.13.2 (statistical tests), and PyRadiomics 3.1.0 (feature extraction).

The reporting quality of this radiomics study was assessed using the following guidelines: the Radiomics Quality Score (RQS) 2.0 [18], the Checklist for Artificial Intelligence in Medical Imaging (CLEAR) [19], and the Machine Learning Evaluation Toolkit in Radiology and Imaging Sciences (METRICS) [20]. The completed checklists are provided in Supplementary Appendices 1, 2, and 3, respectively.

## Results

### Baseline characteristics

Of the 545 patients who underwent NAC for RMS, 7 were excluded from this study because of incomplete clinical data, 23 were excluded because of poor image quality or missing image sequences, 5 were excluded because of treatment history prior to NAC, and 3 were excluded because of concurrent malignancies (Fig. 1). The patients’ characteristics are presented in Table 1. The mean age was 59.00 (34.00–101.00) months, with males constituting 60.31% (313/519) of the cohort. Pathologically, ERMS was the most common (51.64%, 268/519), followed by ARMS (40.66%, 211/519) and sclerosing/spindle cell rhabdomyosarcoma (ScRMS, 7.71%, 40/519). Fusion gene positivity was observed in 28.90% (150/519) of cases. Most tumors were > 5 cm (65.32%, 339/519) and located in unfavorable anatomic sites (defined as parameningeal, extremities, genitourinary bladder/prostate, and other sites; 65.32%, 339/519). Regarding TNM staging, invasive tumors (T2) accounted for 76.69% (398/519), while non-invasive tumors (T1) comprised 23.31% (121/519). Lymph node involvement (N1) was present in 51.25% (266/519) of patients, whereas 48.75% (253/519) had no nodal metastasis (N0). Distant metastasis (M1) was identified in 23.89% (124/519) of cases. No significant differences were observed among the groups for any of the characteristics (*p* > 0.05), indicating balanced distributions across subsets.

**Table 1:** Patients’ clinical characteristics

Variable	Total (*n* = 519)	Training (*n* = 290)	Validation (*n* = 125)	Test 1 (*n* = 61)	Test 2 (*n* = 43)	*p*-value
Age (months), median (IQR)	59.00 (34.00–101.00)	69.50 (39.00–105.00)	56.00 (26.00–101.00)	42.00 (25.00–77.00)	68.00 (40.50–102.50)	0.033
Sex						0.166
Male	313 (60.31)	173 (59.66)	82 (65.60)	30 (49.18)	28 (65.12)	
Female	206 (39.69)	117 (40.34)	43 (34.40)	31 (50.82)	15 (34.88)	
Pathology						0.449
ERMS	268 (51.64)	149 (51.38)	63 (50.40)	30 (49.18)	26 (60.47)	
ARMS	211 (40.66)	115 (39.66)	57 (45.60)	26 (42.62)	13 (30.23)	
ScRMS	40 (7.71)	26 (8.97)	5 (4.00)	5 (8.20)	4 (9.30)	
Fusion_gene						0.423
Negative	369 (71.10)	206 (71.03)	87 (69.60)	41 (67.21)	35 (81.40)	
Positive	150 (28.90)	84 (28.97)	38 (30.40)	20 (32.79)	8 (18.60)	
Primary tumor size						0.921
≤ 5 cm	180 (34.68)	99 (34.14)	43 (34.40)	21 (34.43)	17 (39.53)	
> 5 cm	339 (65.32)	191 (65.86)	82 (65.60)	40 (65.57)	26 (60.47)	
Anatomic site						0.286
Favorable	180 (34.68)	97 (33.45)	39 (31.20)	27 (44.26)	17 (39.53)	
Unfavorable	339 (65.32)	193 (66.55)	86 (68.80)	34 (55.74)	26 (60.47)	
Tumor invasiveness						0.791
T1	121 (23.31)	64 (22.07)	32 (25.60)	16 (26.23)	9 (20.93)	
T2	398 (76.69)	226 (77.93)	93 (74.40)	45 (73.77)	34 (79.07)	
Nodal involvement						0.797
N0	253 (48.75)	141 (48.62)	59 (47.20)	29 (47.54)	24 (55.81)	
N1	266 (51.25)	149 (51.38)	66 (52.80)	32 (52.46)	19 (44.19)	
Metastasis						0.478
M0	395 (76.11)	225 (77.59)	90 (72.00)	49 (80.33)	31 (72.09)	
M1	124 (23.89)	65 (22.41)	35 (28.00)	12 (19.67)	12 (27.91)	
IRS group						0.478
III	395 (76.11)	225 (77.59)	90 (72.00)	49 (80.33)	31 (72.09)	
IV	124 (23.89)	65 (22.41)	35 (28.00)	12 (19.67)	12 (27.91)	

Favorable = orbit, genitourinary nonbladder and prostate and head-neck nonparameningeal; Unfavorable = all other sites (parameningeal, extremities, genitourinary bladder and prostate and other sites)

*ERMS* embryonal rhabdomyosarcoma, *ARMS* alveolar rhabdomyosarcoma, *ScRMS* sclerosing/spindle cell rhabdomyosarcoma, *IRS* Intergroup Rhabdomyosarcoma Study postsurgical grouping system

### Performance of T1CE radiomic models

In the evaluation of T1CE-based radiomic models (Supplementary Tables S5, S6), the T1CE_IntraPeri2mm model demonstrated superior performance across cohorts. This model achieved an AUC of 0.917 (95% CI: 0.8848–0.9483) in the training cohort, with significant superiority over the T1CE_Intra model via DeLong’s test (*p* < 0.001). In the validation cohort, its AUC was 0.760 (95% CI: 0.6736–0.8463), and it exhibited exceptional generalizability in the external test2 cohort: AUC = 0.843 (95% CI: 0.7157–0.9700), sensitivity = 0.667, specificity = 0.857, and F1-score = 0.690. Statistical comparisons (Supplementary Table S6) revealed significant improvements in net reclassification index (NRI = 0.34, *p* < 0.05) and integrated discrimination improvement (IDI = 0.177, *p* < 0.05) for T1CE_IntraPeri2mm compared to baseline models. In contrast, T1CE_Intra showed poor performance in external test2 (AUC = 0.464, 95% CI: 0.2905–0.6381) with a severe sensitivity-specificity imbalance (0.933 vs. 0.250), and was consistently outperformed by T1CE_IntraPeri2mm in pairwise DeLong’s tests (*p* < 0.05). Other candidates (e.g., T1CE_ImageFusion2mm) showed negative or non-significant NRI/IDI improvements in multiple cohorts. Therefore, T1CE_IntraPeri2mm is recommended as the optimal model for the T1CE sequence, balancing predictive accuracy, generalizability, and statistical significance.

### Performance of T2Fs radiomic models

Among the T2Fs-based radiomic models (Supplementary Tables S6, S7), the T2Fs_IntraPeri2mm model demonstrated optimal cross-cohort robustness. It achieved an AUC of 0.928 (95% CI: 0.8985–0.9582) in the training cohort with balanced sensitivity (0.925) and specificity (0.793), yielding an F1-score of 0.810. Notably, its predictive performance improved in external tests: AUC = 0.706 (95% CI: 0.5616–0.8498) in external test1 with sensitivity = 0.636 and specificity = 0.769, and peak performance in test2 (AUC = 0.760, 95% CI: 0.6031–0.9159) with well-balanced metrics (sensitivity = 0.667, specificity = 0.786, F1-score = 0.645). Statistical comparisons (Supplementary Table S7) revealed T2Fs_IntraPeri2mm significantly outperformed T2Fs_ImageFusion2mm in external test2 (DeLong *p* = 0.04; NRI = −0.329, *p* = 0.033; IDI = −0.119, *p* = 0.007), with consistently positive NRI/IDI trends across external cohorts. While T2Fs_ImageFusion2mm showed exceptional training performance (AUC = 0.968), it exhibited significant performance degradation in validation (AUC = 0.598) and external test2 (AUC = 0.558), suggesting overfitting. The T2Fs_Intra model demonstrated sensitivity-specificity imbalance (0.667 vs. 0.500) in test2 with suboptimal AUC = 0.636. Based on cross-cohort generalizability and statistical significance, T2Fs_IntraPeri2mm is recommended as the optimal model for T2Fs sequences.

### Performance of T1CET2Fs radiomic models

Among the T1CET2Fs-derived radiomic models (Supplementary Tables S8, S9), the T1CET2Fs_IntraPeri2mm model demonstrated optimal cross-cohort performance. In the training cohort, it achieved an AUC of 0.927 (95% CI: 0.8965–0.9574; Supplementary Table S8), with non-significant but improved risk stratification over T1CET2Fs_INTRA (DeLong’s *p* = 0.061; NRI = 0.352, *p* = 0.067; IDI = 0.098, *p* = 0.1) (Supplementary Table S9). The model retained stability in validation (AUC = 0.855, 95% CI: 0.7756–0.9340) with balanced sensitivity (0.684) and specificity (0.920), and generalizability in external test cohorts: test1 (AUC = 0.837, 95% CI: 0.7222–0.9515; F1 = 0.762) and test2 (AUC = 0.838, 95% CI: 0.6870–0.9892; specificity = 0.964, PPV = 0.909). By contrast, T1CET2Fs_INTRA exhibited marked overfitting (external test1 AUC = 0.517, 95% CI: 0.3528–0.6810; external test2 AUC = 0.664) and T1CET2Fs_ImageFusion2mm showed critical failure (test1 AUC = 0.500; test2 IDI = −0.201, *p* = 0.001). Statistical comparisons confirmed T1CET2Fs_IntraPeri2mm’s superiority: significant improvements over T1CET2Fs_ImageFusion2mm (NRI = 0.514, *p* = 0.002; IDI = 0.201, *p* = 0.001) and balanced sensitivity-specificity trade-offs versus T1CET2Fs_Peri2mm (external test2 sensitivity = 0.667 vs. 0.867; specificity = 0.964 vs. 0.643). Thus, T1CET2Fs_IntraPeri2mm is recommended for clinical deployment due to its robust generalizability.

### Optimal radiomic models comparison

**Fig. 3:**
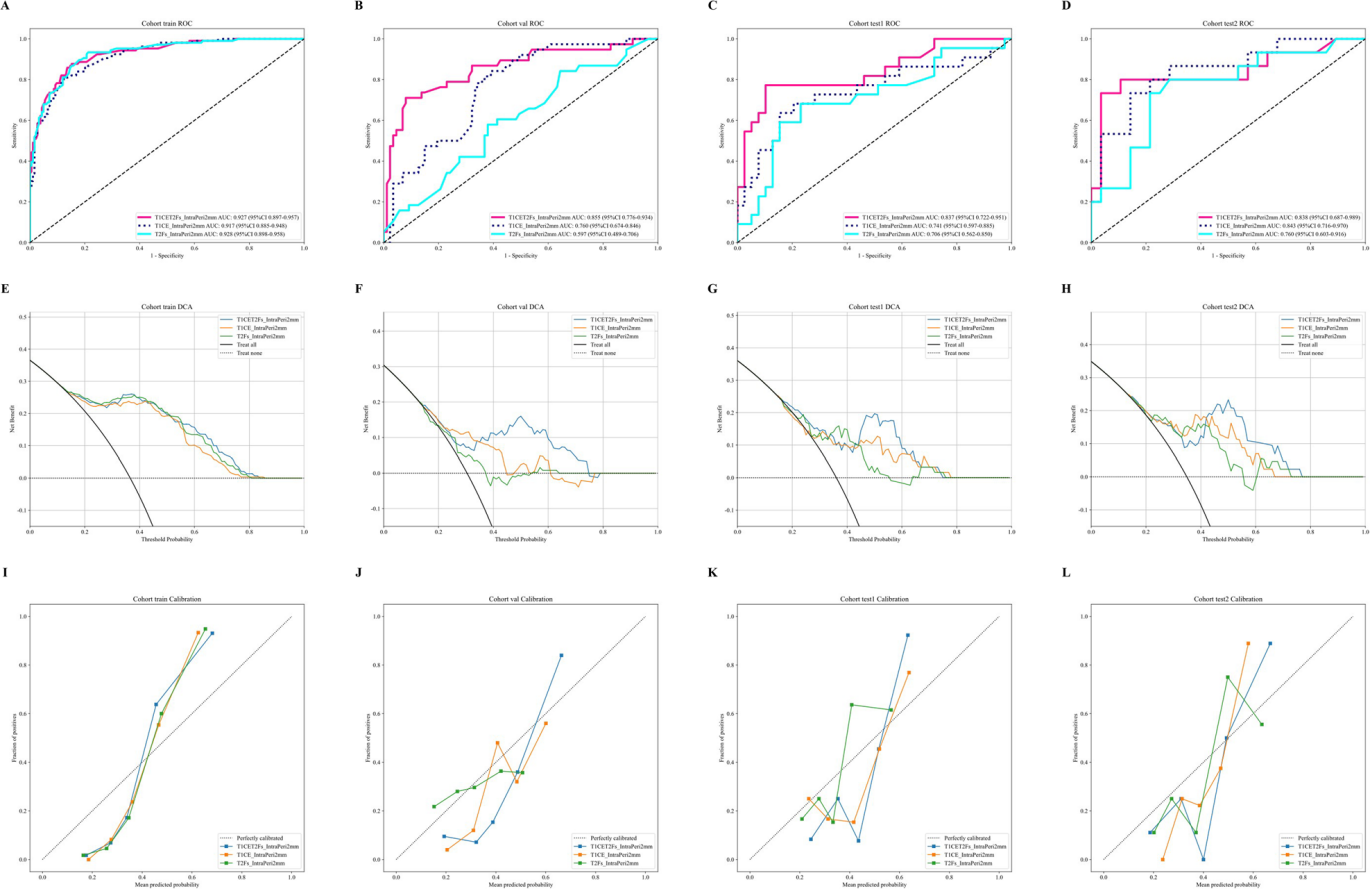
ROC curves: (**A**–**D**), DCA (**E**–**H**), and Calibration curve (**I**–**L**) of the three optimal radiomics models across the training, internal validation, external test1 and external test2 cohorts; ROC, receiver operator characteristic curve; T1CE, T1-weighted contrastenhanced; T2Fs, T2-weighted fat saturated; Val, Validation; DCA, decision curve analysis

In the performance evaluation of T1CE_IntraPeri2mm, T2Fs_IntraPeri2mm, and the multisequence fusion model T1CET2Fs_IntraPeri2mm, the T1CE_IntraPeri2mm model demonstrated superior cross-cohort stability and clinical utility (Fig. 3A–L). While the three models showed comparable AUCs in the training cohort (0.917–0.928; DeLong *p* > 0.05; Table 2), T1CE_IntraPeri2mm achieved the best generalizability in the external test2 cohort (AUC = 0.843, 95% CI: 0.7157–0.9700; Fig. 3D), with the smallest AUC decline from training to external test2 and stable specificity (external test1/test2: 0.846/0.857) and classification thresholds (0.434–0.520; Table 2). Decision curve analysis (DCA) revealed that T1CE_IntraPeri2mm provided the highest net benefit within the clinical decision threshold range (0.2–0.5) in external test2 (Fig. 3H), alongside calibration curves closest to the ideal diagonal (Fig. 3L), outperforming T2Fs_IntraPeri2mm (AUC = 0.760; DeLong *p* = 0.362; Table 3) and the fusion model T1CET2Fs_IntraPeri2mm (abnormally elevated specificity = 0.964 without sensitivity improvement). Cross-cohort AUC curves (Fig. 3A–D) further validated the robustness of T1CE_IntraPeri2mm, while the fusion model showed no significant difference in external test2 AUC (0.838 vs. T1CE, *p* = 0.512; Fig. 3D). Despite integrating multisequence features, the fusion model exhibited no significant improvements in likelihood ratio tests (LRT *p* = 1.0; Table S2), NRI (−0.107, *p* > 0.05), or IDI (−0.056, *p* > 0.05; Table 3). Based on cross-cohort robustness, balanced sensitivity/specificity (external test2: 0.667/0.857), and clinical decision efficacy (Fig. 3D, H, L), T1CE_IntraPeri2mm was identified as the optimal model. Furthermore, subgroup analyses demonstrate that the predictive performance (AUC, sensitivity, specificity) of the T1CE_IntraPeri2mm remains robust and consistent across the pathological phenotypes of RMS (Supplementary Table S10).

**Table 2:** Performance of three optimal models across the three datasets

Model	Accuracy	AUC	95% CI	Sensitivity	Specificity	PPV	NPV	Precision	Recall	F1	Threshold	Cohort
T1CET2Fs_IntraPeri2mm	0.855	0.927	0.8965–0.9574	0.868	0.848	0.767	0.918	0.767	0.868	0.814	0.368	train
T1CE_IntraPeri2mm	0.848	0.917	0.8848–0.9483	0.792	0.88	0.792	0.88	0.792	0.792	0.792	0.434	train
T2Fs_IntraPeri2mm	0.841	0.928	0.8985–0.9582	0.925	0.793	0.721	0.948	0.721	0.925	0.81	0.383	train
T1CET2Fs_IntraPeri2mm	0.848	0.855	0.7756–0.9340	0.684	0.92	0.788	0.87	0.788	0.684	0.732	0.508	val
T1CE_IntraPeri2mm	0.672	0.76	0.6736–0.8463	0.816	0.609	0.477	0.883	0.477	0.816	0.602	0.389	val
T2Fs_IntraPeri2mm	0.6	0.597	0.4892–0.7056	0.553	0.621	0.389	0.761	0.389	0.553	0.457	0.352	val
T1CET2Fs_IntraPeri2mm	0.836	0.837	0.7222–0.9515	0.727	0.897	0.8	0.854	0.8	0.727	0.762	0.499	test1
T1CE_IntraPeri2mm	0.754	0.741	0.5973–0.8852	0.591	0.846	0.684	0.786	0.684	0.591	0.634	0.52	test1
T2Fs_IntraPeri2mm	0.721	0.706	0.5616–0.8498	0.636	0.769	0.609	0.789	0.609	0.636	0.622	0.375	test1
T1CET2Fs_IntraPeri2mm	0.86	0.838	0.6870–0.9892	0.667	0.964	0.909	0.844	0.909	0.667	0.769	0.508	test2
T1CE_IntraPeri2mm	0.791	0.843	0.7157–0.9700	0.667	0.857	0.714	0.828	0.714	0.667	0.69	0.445	test2
T2Fs_IntraPeri2mm	0.744	0.76	0.6031–0.9159	0.667	0.786	0.625	0.815	0.625	0.667	0.645	0.436	test2

*T1CE* T1-weighted contrast-enhanced, *T2Fs* T2-weighted fat-saturated, *Intra* intratumoral regions, *Peri* peritumoral regions, *AUC* area under the receiver operator characteristic curve, *CI* confidence interval, *PPV* positive predictive value, *NPV* negative predictive value, *F1* F1 score

**Table 3:** Comparison of three optimal models using the DeLong’s test, net reclassification improvement, integrated discrimination improvement and likelihood ratio test across the three datasets

Model 1	Model 2	DeLong *p*-value	NRI	NRI 95% CI	NRI *p*-value	IDI	IDI 95% CI	IDI *p*-value	LRT	LRT 95% CI	LRT *p*-value	Cohort
T1CE_IntraPeri2mm	T1CET2Fs_IntraPeri2mm	0.961	−0.107	(−0.461, 0.239)	0.549	−0.056	(−0.158, 0.043)	0.275	−1.908	(−9.759, 5.574)	1	train
T2Fs_IntraPeri2mm	T1CET2Fs_IntraPeri2mm	0.216	−0.179	(−0.476, 0.097)	0.222	−0.061	(−0.141, 0.018)	0.132	−3.994	(−10.487, 1.564)	1	train
T2Fs_IntraPeri2mm	T1CE_IntraPeri2mm	0.362	−0.071	(−0.465, 0.307)	0.717	−0.005	(−0.099, 0.101)	0.921	−2.086	(−9.34, 5.374)	1	train
T1CE_IntraPeri2mm	T1CET2Fs_IntraPeri2mm	0.961	−0.107	(−0.447, 0.241)	0.542	−0.056	(−0.163, 0.044)	0.289	−1.908	(−9.946, 5.43)	1	val
T2Fs_IntraPeri2mm	T1CET2Fs_IntraPeri2mm	0.216	−0.179	(−0.502, 0.124)	0.263	−0.061	(−0.153, 0.02)	0.167	−3.994	(−10.581, 1.352)	1	val
T2Fs_IntraPeri2mm	T1CE_IntraPeri2mm	0.362	−0.071	(−0.472, 0.332)	0.728	−0.005	(−0.105, 0.091)	0.919	−2.086	(−9.539, 5.234)	1	val
T1CE_IntraPeri2mm	T1CET2Fs_IntraPeri2mm	0.961	−0.107	(−0.451, 0.251)	0.55	−0.056	(−0.164, 0.052)	0.31	−1.908	(−9.179, 5.257)	1	test1
T2Fs_IntraPeri2mm	T1CET2Fs_IntraPeri2mm	0.216	−0.179	(−0.472, 0.119)	0.236	−0.061	(−0.151, 0.023)	0.169	−3.994	(−9.977, 1.584)	1	test1
T2Fs_IntraPeri2mm	T1CE_IntraPeri2mm	0.362	−0.071	(−0.478, 0.296)	0.718	−0.005	(−0.109, 0.099)	0.924	−2.086	(−10.473, 5.769)	1	test1
T1CE_IntraPeri2mm	T1CET2Fs_IntraPeri2mm	0.961	−0.107	(−0.439, 0.238)	0.535	−0.056	(−0.156, 0.05)	0.287	−1.908	(−9.301, 5.236)	1	test2
T2Fs_IntraPeri2mm	T1CET2Fs_IntraPeri2mm	0.216	−0.179	(−0.498, 0.135)	0.269	−0.061	(−0.144, 0.019)	0.142	−3.994	(−9.801, 2.053)	1	test2
T2Fs_IntraPeri2mm	T1CE_IntraPeri2mm	0.362	−0.071	(−0.463, 0.327)	0.723	−0.005	(−0.1, 0.089)	0.916	−2.086	(−9.685, 5.058)	1	test2

*T1CE* T1-weighted contrast-enhanced, *T2Fs* T2-weighted fat-saturated, *Intra* intratumoral regions, *Peri* peritumoral regions, *NRI* net reclassification improvement, *CI* confidence interval, *IDI* integrated discrimination improvement, *LRT* likelihood ratio test

### Combined clinical-radiomic model

The combined clinical-radiomic model (Combined) demonstrated no statistically or clinically significant incremental benefit over the standalone radiomic model (T1CE_IntraPeri2mm) (Supplementary Tables S11, S12). In the external test2 cohort, the Combined model showed negligible AUC improvement (0.843 vs. 0.838; DeLong’s *p* = 0.891) and failed to optimize sensitivity-specificity equilibrium (0.667 vs. 0.667 sensitivity, 0.857 vs. 0.964 specificity), with non-significant F1-score changes (0.690 vs. 0.769) (Supplementary Table S10). Statistical tests revealed no significant enhancements in net reclassification (NRI = 0.01, *p* = 0.754) or integrated discrimination (IDI = 0.003, *p* = 0.814) for the Combined model (Supplementary Table S11). Notably, the Combined model exhibited a larger AUC decline from training to test2 and higher threshold volatility, suggesting potential overfitting from feature fusion.

### Model interpretation

**Fig. 4:**
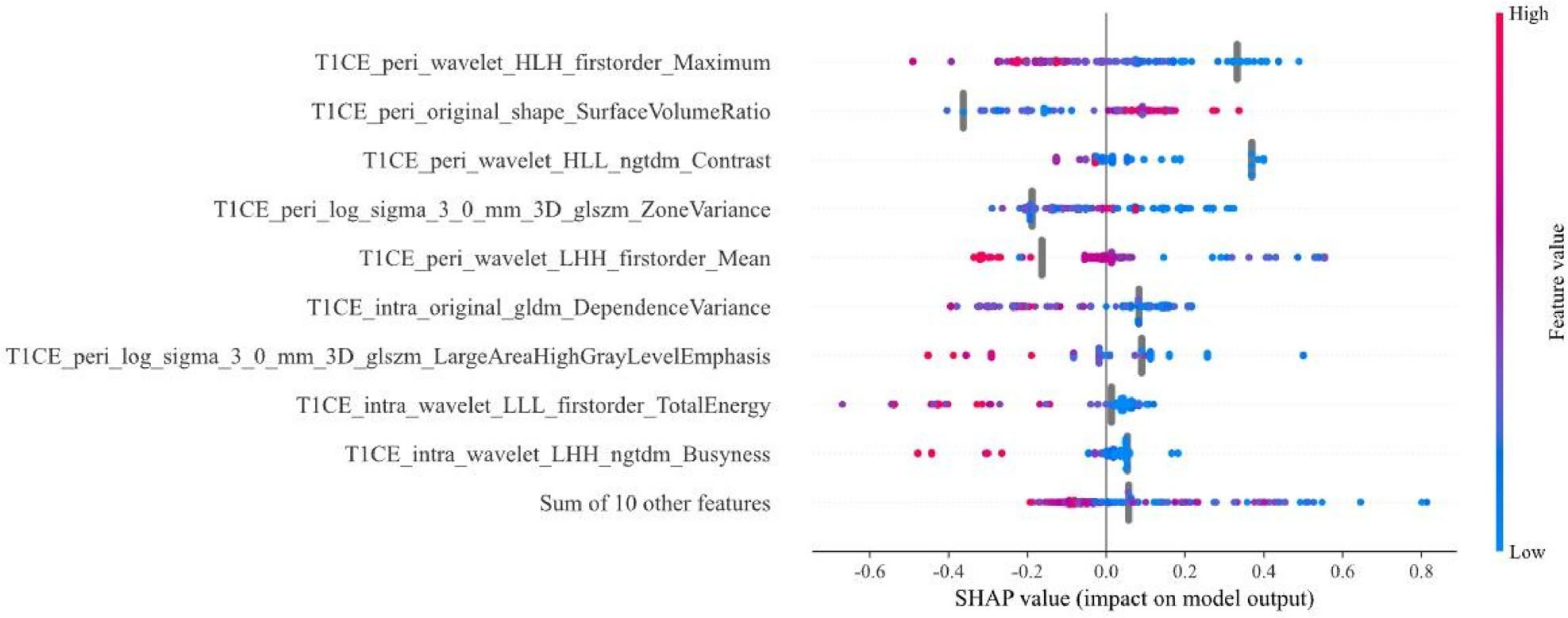
SHAP summary plots of the T1CE_IntraPeri2mm model. T1CE, T1-weighted contrast-enhanced; peri, peritumoral regions; HLH, high-low-high; HLL, high-low-low; ngtdm, neighborhood gray-tone difference matrix; glszm, gray-level size zone matrix; LHH, low-high-high; intra, intratumoral regions; gldm, gray-level dependence matrix; LLL, low-low-low

SHAP interpretability analysis elucidated the differential contributions of peritumoral and intratumoral radiomic features to chemotherapy response prediction (Fig. 4). Among peritumoral features, high values of T1CE_peri_wavelet_HLH_firstorder_Maximum (maximum intensity in wavelet-transformed peri-tumor regions on contrast-enhanced T1-weighted imaging) exhibited significantly negative SHAP values, indicating an association between expanded high-intensity peritumoral regions and chemotherapy sensitivity. Conversely, high values of T1CE_peri_original_shape_SurfaceVolumeRatio (surface-area-to-volume ratio of peri-tumor morphology) showed positive SHAP contributions, suggesting that irregular peritumoral structural patterns were linked to chemoresistance. For intratumoral features, high values of T1CE_intra_wavelet_LHH_ngtdm_Busyness (local intensity busyness in wavelet-transformed intra-tumor regions) demonstrated negative SHAP effects, reflecting their potential biological relevance to chemotherapy sensitivity. Notably, peritumoral heterogeneity metrics T1CE_peri_wavelet_HLL_ngtdm_Contrast and T1CE_peri_log_sigma_3_0_mm_3D_glszm_ZoneVariance displayed nonlinear threshold effects, implying complex interactions with treatment response that may depend on microenvironmental immune status. These findings validate the multi-scale decision logic of the model: both peritumoral structural features (morphology/signal distribution) and intratumoral heterogeneity jointly drive chemotherapy response prediction.

## Discussion

The present multicenter study represents the largest radiomics investigation to date in pediatric RMS, systematically evaluating the predictive value of both intratumoral and peritumoral MRI features for NAC response. While previous radiomic studies in STS have demonstrated promising predictive capabilities for NAC response [10, 17, 21], models tailored to specific histologic subtypes remain scarce. This gap is particularly critical given the extreme heterogeneity of STS, which encompasses over 100 distinct entities in the WHO 2020 classification [22]. As RMS constitutes 50% of pediatric STS cases in children aged 0–14 years [2], our subtype-specific analysis addresses a vital unmet need. Our findings demonstrate that radiomic models integrating peritumoral microenvironment characteristics, particularly those derived from T1CE sequences, achieve superior predictive performance compared to conventional intratumoral or clinical models. The optimal T1CE_IntraPeri2mm model demonstrated robust cross-cohort generalizability (AUC: 0.760–0.917), outperforming multisequence fusion approaches and providing critical insights into the biological interplay between tumor heterogeneity and treatment resistance. These results align with emerging evidence across oncology that peritumoral radiomic features capture microenvironmental alterations critical to therapeutic response [23–25].

The observed predictive advantage of peritumoral-enhanced models suggests potential biological links to tumor-stroma interactions [26]. SHAP analysis revealed that peritumoral wavelet-transformed maximum intensity (T1CE_peri_wavelet_HLH_firstorder_Maximum) and surface-area-to-volume ratio (T1CE_peri_original_shape_SurfaceVolumeRatio) were key predictors, suggesting that both metabolic activity and morphological irregularity in the tumor periphery influence treatment outcomes. Their synergistic predictive power implies that therapeutic resistance arises not only from treatment evasion driven by peritumoral hypermetabolism but also from potentially compromised drug penetration and inadequate radiation coverage due to irregular boundary geometry. This discovery could inform future research on personalized therapeutic strategies.

Multisequence analysis reveals limitations of feature fusion strategies in RMS. Despite theoretical complementarity between T1CE and T2Fs sequences, the fused model T1CET2Fs_IntraPeri2mm showed no significant superiority over single-sequence models in the external test, contradicting findings in glioblastoma [27] and breast cancer [28]. We think the comparable performance of T1CE-based models versus fused models stems from: (1) Biological redundancy—T1CE’s contrast mechanisms encode key therapeutic biomarkers; (2) Noise interference—T2Fs’ nonspecific pathological signals; (3) Dimensional inflation—Feature concatenation increases complexity without proportional information gain, risking overfitting in RMS’s limited cohorts. These findings align with Lambin’s caution against indiscriminate multiparametric fusion [29], advocating biologically-driven sequence selection. The consistent superiority of T1WI (particularly contrast-enhanced) resonates with STS radiomics consensus: Core sequences (e.g., T1CE, T2FS) excel in delineating tumor boundaries and heterogeneity [21, 30]. For instance, Crombé et al predicted STS chemotherapy response using delta-radiomics from enhanced T1WI/T2WI [30], while Peeken et al combined T1CE-FS/T2FS for high-grade STS outcome prediction [31]. However, excessive multisequence integration risks compounding protocol standardization challenges due to inherent differences in MRI contrast mechanisms (T1 relaxation vs. T2 proton density) and spatial resolution, particularly problematic in rare pediatric tumors like RMS, where dimensionality curse emerges (e.g., Fields et al achieved AUC = 0.44 using 11 sequences in 44 patients) [21]. Thus, prioritizing biologically relevant single sequences (e.g., T1CE) enhances model robustness while balancing clinical efficiency and patient comfort in pediatric imaging, offering critical guidance for optimizing radiomics paradigms.

The T1CE_IntraPeri2mm model established in this study demonstrates significant clinical utility in predicting NAC response for RMS. With a specificity of 0.857 in identifying non-responders using routine MRI sequences, the model enables risk-adapted therapeutic stratification, allowing early termination of ineffective chemotherapy for resistant patients while intensifying tumor control for sensitive subgroups [3, 32]. This stratification principle aligns with the Children’s Oncology Group (COG) clinical trial framework [33, 34], proving crucial for advancing precision medicine in RMS. Notably, although the radiomics model outperforms conventional volumetric assessment, its moderate sensitivity necessitates future integration of liquid biopsies or molecular biomarkers (e.g., PAX-FOXO1 fusion status) to optimize predictive capacity [35–37]. The clinical significance is further amplified by the unique challenges in RMS therapeutic evaluation. Distinct from adult soft-tissue sarcomas using pathological complete response (pCR) as endpoints [8, 31], RMS management requires a careful balance between tumor control and long-term quality of life in pediatric patients [5, 38]. Conventional volumetric assessment often fails due to tumor necrosis heterogeneity, cytostatic effects, and pseudoprogression [11, 39]. Our model overcomes these limitations through multiparametric feature extraction, enabling reliable early stratification during NAC—particularly valuable for tumors in complex anatomical sites (skull base, orbit) where delayed resection is impractical [40, 41]. While previous studies reported inconsistent correlations between early volume reduction and survival outcomes [42–44], the high specificity of our model provides a critical time window for therapeutic modification: early transition to radiotherapy/surgery for resistant cases [45], and NAC intensification for responders to maximize tumor control. Intriguingly, the clinical-radiomics integrated model showed no performance improvement, contrasting with adult sarcoma studies where clinical parameters (tumor size, location) enhanced model efficacy [46]. This discrepancy may stem from the distinct biology of pediatric RMS, where molecular characteristics (e.g., fusion gene status) outweigh conventional clinical factors in prognostic dominance [37]. Our findings underscore the necessity of age-specific biological rationale in pediatric prediction model development, urging future research to focus on multi-omics integration strategies.

This study has several limitations that warrant discussion. First, while the multicenter design and large sample size enhance generalizability, the retrospective nature introduces inherent selection bias that may influence feature reproducibility. Second, manual tumor segmentation, while reviewed by senior radiologists, remains time-consuming and subjective compared to automated approaches—future integration of deep learning segmentation could enhance reproducibility. Third, given the controversy surrounding the correlation between NAC response and survival outcomes in RMS [33, 34], our focus on early volumetric response (post-cycle 3) rather than survival endpoints necessitates caution in clinical interpretation. Fourth, the exclusion of functional MRI sequences (diffusion-weighted imaging/dynamic contrast-enhanced, DWI/DCE) limits direct comparison with emerging radiomic biomarkers derived from multiparametric protocols. Finally, the biological plausibility of identified peritumoral features requires spatial validation through techniques like spatially resolved transcriptomics or multiplex immunohistochemistry mapping stromal immune infiltration.

In conclusion, this multicenter study establishes peritumoral radiomics from routine T1CE MRI as a robust predictor of NAC response in pediatric RMS, overcoming limitations of conventional clinical assessment. By decoding the radiologic signature of tumor-microenvironment interactions, our findings provide a foundation for image-guided personalized therapy in this vulnerable population, while underscoring the necessity of incorporating age-specific biological rationale into pediatric prediction models.

## ELECTRONIC SUPPLEMENTARY MATERIAL

Supplementary information

## Data Availability

The in-house data supporting the findings of this study are available from the corresponding author upon reasonable request.

## Abbreviations

AUC: Area under the receiver operating characteristic curve
ARMS: Alveolar rhabdomyosarcoma
BCH: Beijing Children’s Hospital
CI: Confidence interval
DCA: Decision curve analysis
ERMS: Embryonal rhabdomyosarcoma
ICC: Intraclass correlation coefficient
IDI: Integrated discrimination improvement
IRS: Intergroup rhabdomyosarcoma study
LASSO: Least absolute shrinkage and selection operator
LRT: Likelihood ratio test
MRI: Magnetic resonance imaging
NAC: Neoadjuvant chemotherapy
NGTDM: Neighboring gray-tone difference matrix
NRI: Net reclassification improvement
PyRadiomics: Python radiomics library
RMS: Rhabdomyosarcoma
ROI: Region of interest
SHAP: SHapley Additive exPlanations
SMCH: Shunyi Maternal and Children’s Hospital
STS: Soft-tissue sarcoma
T1CE: T1-weighted contrast-enhanced
T2Fs: T2-weighted fat-saturated
TRH: Beijing Tongren Hospital
XGBoost: Extreme Gradient Boosting
