# Supplementary material for: Intratumoral and peritumoral radiomics for the pretreatment prediction of response to neoadjuvant chemotherapy in rhabdomyosarcoma: a multicenter retrospective cohort study
Source: Insights Imaging. 2026 Jan 5;17:3. doi: 10.1186/s13244-025-02178-0 (PMC12770143; doi:10.1186/s13244-025-02178-0)
Supplement: Supplementary file 1 — Supplementary information [file 13244_2025_2178_MOESM1_ESM.pdf]

Intratumoral and Peritumoral Radiomics for the Pretreatment Prediction of Response to Neoadjuvant Chemotherapy in Rhabdomyosarcoma: A Multicenter Retrospective Cohort Study

ELECTRONIC SUPPLEMENTARY MATERIAL

Supplemental Methods 2
Supplemental Figures 6
Figure S1 Selected Features and Their Coefficients of the T1CE, T2Fs, and T1CET2Fs .. 6
Figure S2 Line chart depicting the AUC performance of four machine learning algorithms across 12 feature groups within three datasets 7
Figure S3 Line chart depicting the AUC performance of four machine learning algorithms within Clinic model across three datasets 8
Supplemental Tables 9
Table S1 MRI acquisition parameters across multiple medical centers 9
Table S2 Extracted radiomics features 10
Table S3 Univariate and multivariable logistic regression analyses for selecting clinical features of model development 15
Table S4 Performance of different T1CE models across the three datasets 16
Table S5 Comparison of T1CE models using the Delong test, Net Reclassification Improvement and Integrated Discrimination Improvement across the three datasets 17
Table S6 Performance of different T2Fs models across the three datasets 19
Table S7 Comparison of T2Fs models using the Delong test, Net Reclassification Improvement and Integrated Discrimination Improvement across the three datasets 20
Table S8 Performance of different T1CET2Fs models across the four datasets 21
Table S9 Comparison of T1CET2Fs models using the Delong test, Net Reclassification Improvement, Integrated Discrimination Improvement and likelihood ratio test across the four datasets 22
Table S10 Pathology subgroup analyses of T1CET2Fs_IntraPeri2mm model across the four datasets 23
Table S11 Performance of the Clinic model, the T1CE_IntraPeri2mm model, and the combined model across the four datasets 24
Table S12 Comparison of the Clinic model, the T1CE_IntraPeri2mm model, and the combined model using the Delong test, Net Reclassification Improvement, Integrated Discrimination Improvement and likelihood ratio test across the three datasets 25
Appendix1: Radiomics Quality Score (RQS) 2.0 with Radiomics Readiness Levels (RRLs) 26
Appendix2: CheckList for EvaluAtion of Radiomics research (CLEAR) 32
Appendix3: METhodological RadiomICs Score (METRICS) 34

).

Supplemental Methods

Sample Size Calculation

To ensure the methodological rigor and scientific validity of our radiomics prediction model, we performed a sample size calculation based on the established framework by Riley et al. (Statistics in Medicine, 2018) for binary outcome prediction models. This analysis was conducted to evaluate whether our cohort size (n = 519) provided sufficient statistical power to develop a robust multivariable model while minimizing overfitting and ensuring precise parameter estimation. The calculation adhered to three fundamental criteria recommended for prediction model studies.

The first criterion targeted minimization of overfitting by achieving a global shrinkage factor (S_VH) of at least 0.9, calculated using the formula:

$$n = \frac{p}{(S_{VH} - 1)\ln\left(1 - \frac{R^2_{CS\_adj}}{S_{VH}}\right)}$$

where p represents the number of predictor parameters and R^2_CS_adj denotes the adjusted Cox-Snell R^2 value.

The second criterion ensured limited optimism in model performance by maintaining ≤0.05 absolute difference between apparent and adjusted Nagelkerke's R^2 values, derived from the inequality:

$$S_{VH} \geq 1 - \frac{\delta \times \max(R^2_{CS\_app})}{R^2_{CS\_adj}}$$

with δ set at 0.05.

The third criterion guaranteed precise estimation of the overall outcome risk with a margin of error ≤0.05, approximated by:

$$n \geq \frac{1.96^2 \times \phi(1 - \phi)}{0.05^2}$$

where ϕ indicates the outcome proportion.

Parameter selection was based on conservative estimates from our study data: p = 20 (reflecting typical predictor parameters in radiomics feature selection), R^2_CS_adj = 0.15 (estimated AUC performance of the optimal model in the external test set is 0.8-0.85), ϕ = 0.5 (assuming balanced response distribution), and max(R^2_CS_app) = 0.75 (calculated for ϕ = 0.5).

The analysis revealed that our sample size met the requirements for precise risk estimation (criterion 3, requiring n ≥ 385) and R^2 optimism control (criterion 2). For the shrinkage factor

criterion (criterion 1), the calculated requirement was n = 1097 under conservative parameters, though sensitivity analysis showed this reduced to n = 548 with more optimistic parameters (p = 10). Overall, our sample size is sufficient for model building.

The evaluation criteria for the inclusion and response of enrolled patients

The inclusion criteria were as follows: (a) RMS staged as Intergroup Rhabdomyosarcoma Study postsurgical grouping system (IRS)-III/IV prior to NAC; (b) availability of baseline magnetic resonance imaging; (c) completion of comprehensive therapy. The exclusion criteria encompassed: (a) incomplete clinical data; (b) poor image quality or missing image sequences; (c) any treatment history prior to NAC; (d) concurrent malignancies. Demographic information, morphological and molecular pathological findings, and clinical characteristics were extracted from electronic medical records. For patients with measurable disease (IRS-III/IV), tumor volume at diagnosis and post-three chemotherapy cycles (spanning 7 to 10 weeks) was estimated by measuring the maximum diameter of the primary tumor in three dimensions (X, Y, and Z), using the formula $V=\pi/6X*Y*Z$. This method was employed to assess early response to NAC. With reference to the criteria established in the Children's Oncology Group (COG) study ARST0531 (NCT00354835; 2006–2012)[1], treatment responses are classified as follows: Complete Response (CR), indicating complete tumor disappearance; Partial Response (PR), indicating at least a 64% decrease in volume compared to baseline; Stable Disease/No Response (SD/NR), indicating less than a 64% decrease and no more than a 39% increase in volume; and Progressive Disease (PD), indicating an increase in volume of 40% or more. All response must last at least 4 weeks without evidence of tumour progression or relapse. Objective response rate (ORR) was widely demonstrated for assessing the treatment response, the proportion of patients with PR and CR in all patients was defined as ORR[2]. Therefore, we included patients with PR/CR as the responding group and patients with PD/SD as the non-responding group. Additionally, we categorized CR and PR into the NAC-sensitive group, and SD/NR and PD into the NAC-resistant group.

Manual delineation of tumor and automatic generation of peritumorial

Two radiologists with 10 years of experience in pediatric solid tumor imaging manually delineated the Region of Interest (ROI) of the tumor on all slices of each MRI sequence using ITK-SNAP software (version 3.8.0) and saved it in NIFTI format. To ensure the accuracy of the segmentation, all segmentation results were reviewed and revised by a senior radiologist with 25 years of experience. The radiologists were blinded to all clinical information regarding the patients throughout the tumor segmentation process. Subsequently, the radiomic features of the tumor regions in the various sequence images were extracted based on the delineated

mask. A 2 mm peritumor region was generated by automatically expanding the lesion boundaries, and the intratumoral area was removed. And manual adjustments were made to exclude non-peritumoral areas, such as normal structural tissues like blood vessels, nerves, muscles, bones, as well as empty spaces.

Feature extraction and selection process

Features were derived from the original images and filtered images, including wavelet transforms and Laplacian of Gaussian (LoG) filters. The extracted features encompassed shape, first-order statistics, and texture features derived from gray-level co-occurrence matrices (GLCM), gray-level run-length matrices (GLRLM), gray-level size zone matrices (GLSZM), gray-level dependence matrices (GLDM), and neighboring gray-tone difference matrices (NGTDM). Using feature concatenation technology, we systematically integrated intratumoral features, peritumoral features, and multi-sequence imaging features, merging them into a unified high-dimensional feature vector. A total of 12 feature groups were constructed: Based on the T1CE sequence, four feature sets were generated—intratumoral region features (T1CE_Intra), peritumoral 2mm region features (T1CE_Peri2mm), features from concatenation and selection of intra- and peri-tumoral features (T1CE_IntraPeri2mm), and features extracted from image fusion of intra- and peri-tumoral regions (T1CE_ImageFusion2mm). Similarly, four corresponding feature sets were created from the T2FS sequence. Finally, through cross-sequence integration, similar feature types from T1CE and T2FS were concatenated and refined to form four cross-sequence feature groups: T1CET2Fs_Intra, T1CET2Fs_Peri2mm, T1CET2Fs_IntraPeri2mm, and T1CET2Fs_ImageFusion2mm.

The radiomics feature selection process was conducted as follows: First, feature robustness was evaluated through test-retest and inter-rater analyses. For the test-retest analysis, tumor subregions of 30 randomly selected patients from the discovery dataset were segmented twice by a single rater, while for the inter-rater analysis, tumor subregions of another 30 randomly selected patients were independently segmented by two raters. Features extracted from these multiple segmentations were assessed using the intraclass correlation coefficient (ICC), and only features with ICC ≥ 0.80 were retained to ensure robustness against intra- and inter-rater segmentation uncertainties. Next, all retained features were normalized using Z-score normalization, and their statistical significance was evaluated using an independent t-test, with features exhibiting a p-value < 0.05 being selected. To reduce redundancy and mitigate collinearity, Pearson correlation coefficients were calculated for each feature pair, and one feature from any pair with a correlation coefficient ≥ 0.90 was excluded. Finally, Lasso regression with 10-fold cross-validation was applied to further refine the feature set by identifying the optimal regularization parameter λ, retaining only the most predictive and

informative features with non-zero coefficients. This multi-step approach ensured the selection of stable, significant, and non-redundant features for downstream analysis.

Reference

1. Lautz TB, Chi Y, Tian J, et al (2020) Relationship between tumor response at therapy completion and prognosis in patients with group III rhabdomyosarcoma: a report from the children’s oncology group. Int J Cancer 147:1419–1426. https://doi.org/10.1002/ijc.32896

2. Bradbury P, Seymour L (2009) Tumor shrinkage and objective response rates: gold standard for oncology efficacy screening trials, or an outdated end point? Cancer J 15:354–360. https://doi.org/10.1097/PPO.0b013e3181b9c506

Supplemental Figures

Figure S1 Selected Features and Their Coefficients of the T1CE, T2Fs, and T1CET2Fs

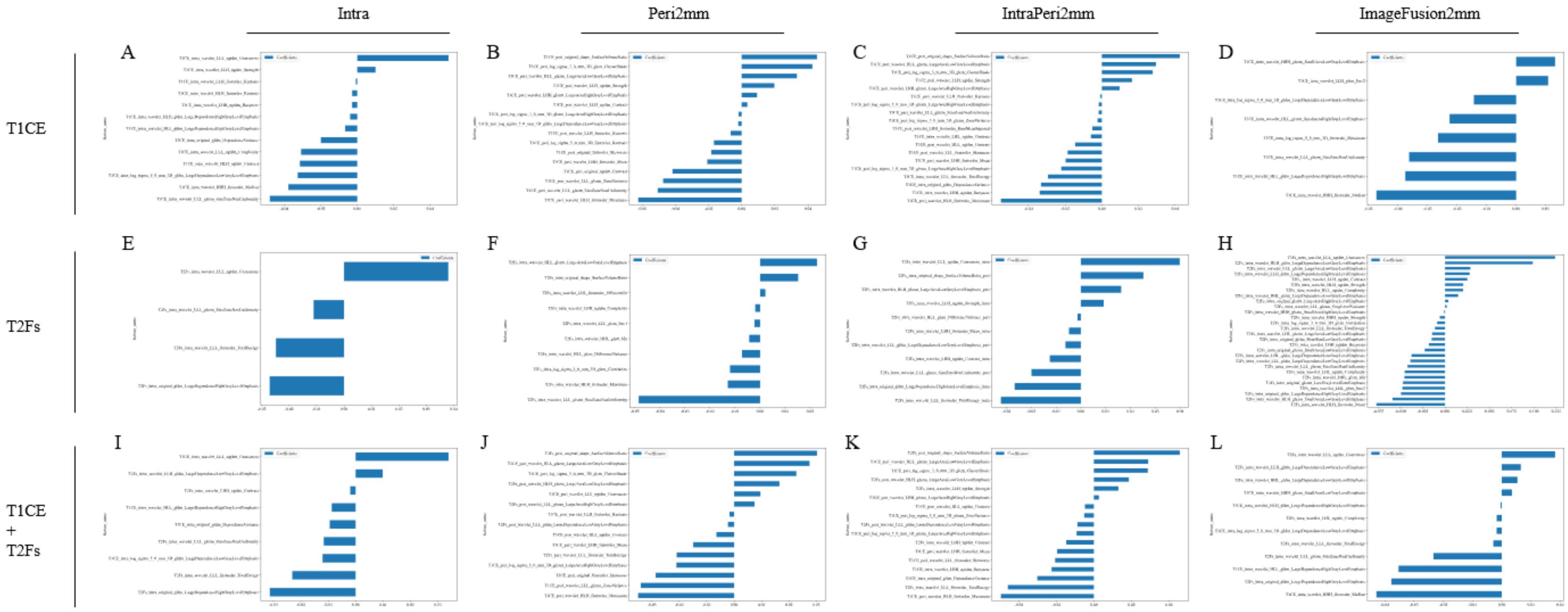

Abbreviation: T1CE, T1-weighted contrast-enhanced; T2Fs, T2-weighted fat saturated, Intra, Intratumoral regions; Peri, Peritumoral regions.

Figure S2 Line chart depicting the AUC performance of four machine learning algorithms across 12 feature groups within three datasets

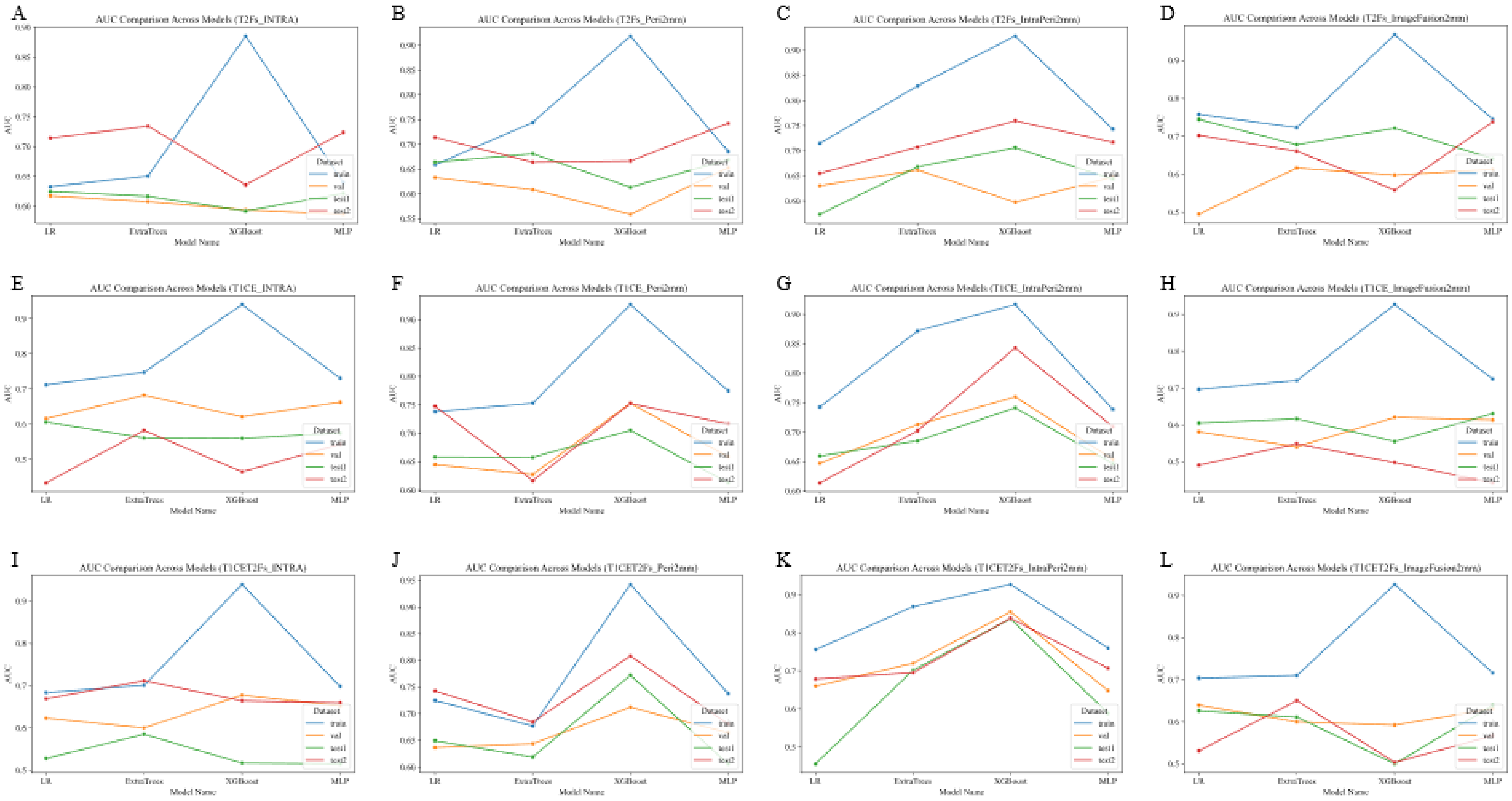

Abbreviation: AUC, Area under the receiver operator characteristic curve; T1CE, T1-weighted contrast-enhanced; T2Fs, T2-weighted fat saturated; Intra, Intratumoral regions; Peri, Peritumoral regions; LR, Logistic regression; XGBoost, Extreme gradient boosting; MLP, Multi-layer perceptron; val, validation.

Figure S3 Line chart depicting the AUC performance of four machine learning algorithms within Clinic model across three datasets

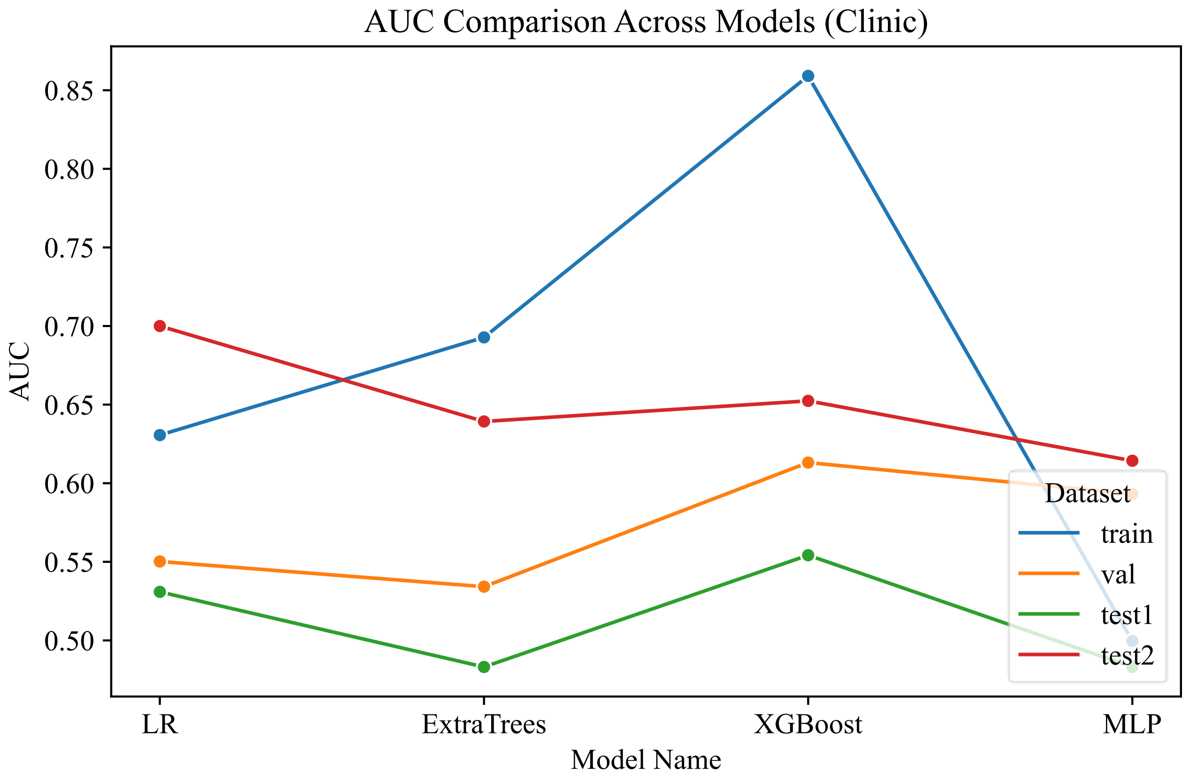

Abbreviation: AUC, Area under the receiver operator characteristic curve; T1CE, T1-weighted contrast-enhanced; T2Fs, T2-weighted fat saturated; Intra, Intratumoral regions; Peri, Peritumoral regions; LR, Logistic regression; XGBoost, Extreme gradient boosting; MLP, Multi-layer perceptron; val, validation.

Supplemental Tables

Table S1 MRI acquisition parameters across multiple medical centers

Cohort	Scanner	Sequence	TR (ms)	TE (ms)	FOV (mm)	Matrix	Thickness (mm)
BCH	GE Discovery MR 750	T1CE	400-600	10-15	300-360	256×192	5
		T2Fs	2000-4000	80-100	300-360	256×224	5
TRH	Siemens Skyra	T1CE	500-700	10-15	280-340	320×240	4
		T2Fs	3000-5000	90-110	280-340	320×256	4
SMCH	GE Signa HDxt	T1CE	450-650	15-25	320-380	256×192	5
		T2Fs	2500-4500	85-105	320-380	256×224	5

Abbreviation: BCH: Beijing Children's Hospital; TRH: Beijing Tongren Hospital; SMCH: Shunyi Maternal and Children’s Hospital of Beijing Children’s Hospital; T1CE: T1-weighted contrast-enhanced; T2Fs: T2-weighted fat saturated; TR: repetition time; TE: echo time; FOV: field of view.

Table S2 Extracted radiomics features

Sequence	Model	Selected features
T1CE	Intra	T1CE_intra_wavelet_LLL_glszm_SizeZoneNonUniformity
		T1CE_intra_wavelet_LLL_ngtdm_Coarseness
		T1CE_intra_wavelet_LHH_ngtdm_Busyness
		T1CE_intra_wavelet_HHH_firstorder_Median
		T1CE_intra_wavelet_LLH_firstorder_Kurtosis
		T1CE_intra_original_gldm_DependenceVariance
		T1CE_intra_wavelet_HLH_firstorder_Kurtosis
		T1CE_intra_log_sigma_5_0_mm_3D_gldm_LargeDependenceLowGrayLevelEmphasis
		T1CE_intra_wavelet_HLL_gldm_LargeDependenceHighGrayLevelEmphasis
		T1CE_intra_wavelet_HLH_ngtdm_Contrast
		T1CE_intra_wavelet_LLL_ngtdm_Complexity
		T1CE_intra_wavelet_HLH_gldm_LargeDependenceHighGrayLevelEmphasis
		T1CE_intra_wavelet_LLH_ngtdm_Strength
	Peri2mm	T1CE_peri_log_sigma_3_0_mm_3D_glcm_ClusterShade
		T1CE_peri_wavelet_LHH_firstorder_Mean
		T1CE_peri_original_shape_SurfaceVolumeRatio
		T1CE_peri_wavelet_HLH_firstorder_Maximum
		T1CE_peri_wavelet_HLL_glszm_LargeAreaLowGrayLevelEmphasis
		T1CE_peri_wavelet_LHH_glszm_LargeAreaHighGrayLevelEmphasis
		T1CE_peri_wavelet_LLH_firstorder_Kurtosis
		T1CE_peri_wavelet_LLL_glszm_SizeZoneNonUniformity
		T1CE_peri_log_sigma_5_0_mm_3D_gldm_LargeDependenceLowGrayLevelEmphasis
		T1CE_peri_wavelet_LLH_ngtdm_Contrast
		T1CE_peri_wavelet_LLL_glszm_ZoneVariance
		T1CE_peri_log_sigma_5_0_mm_3D_firstorder_Kurtosis
		T1CE_peri_original_firstorder_Skewness
		T1CE_peri_wavelet_LLH_ngtdm_Strength
		T1CE_peri_log_sigma_3_0_mm_3D_glszm_LargeAreaHighGrayLevelEmphasis
		T1CE_peri_original_ngtdm_Contrast
	IntraPeri2mm	T1CE_peri_log_sigma_3_0_mm_3D_glcm_ClusterShade
		T1CE_peri_log_sigma_3_0_mm_3D_glszm_ZoneVariance
		T1CE_peri_wavelet_LHH_firstorder_Mean
		T1CE_peri_wavelet_HLL_ngtdm_Contrast
		T1CE_peri_wavelet_LLL_firstorder_Skewness
		T1CE_peri_wavelet_HLL_glszm_LargeAreaLowGrayLevelEmphasis
		T1CE_intra_wavelet_LHH_ngtdm_Busyness
		T1CE_peri_wavelet_HLH_firstorder_Maximum
		T1CE_peri_original_shape_SurfaceVolumeRatio
		T1CE_peri_log_sigma_5_0_mm_3D_glszm_LargeAreaHighGrayLevelEmphasis
		T1CE_intra_original_gldm_DependenceVariance

		T1CE_intra_wavelet_LLL_firstorder_TotalEnergy
		T1CE_peri_wavelet_LHH_glszm_LargeAreaHighGrayLevelEmphasis
		T1CE_intra_wavelet_LHL_ngtdm_Contrast
		T1CE_peri_wavelet_LLL_glszm_SizeZoneNonUniformity
		T1CE_peri_wavelet_LHH_firstorder_RootMeanSquared
		T1CE_peri_wavelet_LLH_firstorder_Kurtosis
		T1CE_peri_log_sigma_3_0_mm_3D_glszm_LargeAreaHighGrayLevelEmphasis
		T1CE_peri_wavelet_LLH_ngtdm_Strength
	ImageFusion 2mm	T1CE_intra_wavelet_HHH_firstorder_Median
		T1CE_intra_wavelet_HHH_glszm_SmallAreaLowGrayLevelEmphasis
		T1CE_intra_wavelet_LLL_glszm_SizeZoneNonUniformity
		T1CE_intra_log_sigma_5_0_mm_3D_gldm_LargeDependenceLowGrayLevelEmphasis
		T1CE_intra_wavelet_HLL_gldm_LargeDependenceHighGrayLevelEmphasis
		T1CE_intra_wavelet_HLL_glszm_SmallAreaHighGrayLevelEmphasis
		T1CE_intra_wavelet_LLH_glcm_Imc2
		T1CE_intra_log_sigma_5_0_mm_3D_firstorder_Maximum
T2Fs	Intra	T2Fs_intra_wavelet_LLL_glszm_SizeZoneNonUniformity
		T2Fs_intra_wavelet_LLL_firstorder_TotalEnergy
		T2Fs_intra_original_gldm_LargeDependenceHighGrayLevelEmphasis
		T2Fs_intra_wavelet_LLL_ngtdm_Coarseness
	Peri2mm	T2Fs_intra_wavelet_HLL_glcm_DifferenceVariance
		T2Fs_intra_original_shape_SurfaceVolumeRatio
		T2Fs_intra_wavelet_LLL_glszm_SizeZoneNonUniformity
		T2Fs_intra_log_sigma_3_0_mm_3D_glcm_Correlation
		T2Fs_intra_wavelet_HLL_glszm_LargeAreaLowGrayLevelEmphasis
		T2Fs_intra_wavelet_HLH_firstorder_Maximum
		T2Fs_intra_wavelet_LHL_firstorder_10Percentile
		T2Fs_intra_wavelet_HHL_glcm_Idn
		T2Fs_intra_wavelet_LHH_ngtdm_Complexity
		T2Fs_intra_wavelet_LLL_glcm_Imc1
	IntraPeri2mm	T2Fs_intra_original_shape_SurfaceVolumeRatio_peri
		T2Fs_intra_wavelet_LLL_gldm_LargeDependenceLowGrayLevelEmphasis_peri
		T2Fs_intra_wavelet_LHH_ngtdm_Contrast_intra
		T2Fs_intra_wavelet_LLH_ngtdm_Strength_intra
		T2Fs_intra_wavelet_HLL_glcm_DifferenceVariance_peri
		T2Fs_intra_wavelet_HLH_glszm_LargeAreaLowGrayLevelEmphasis_peri
		T2Fs_intra_wavelet_LLL_firstorder_TotalEnergy_intra
		T2Fs_intra_wavelet_LHH_firstorder_Mean_intra
		T2Fs_intra_wavelet_LLL_ngtdm_Coarseness_intra
		T2Fs_intra_wavelet_LLL_glszm_SizeZoneNonUniformity_peri

		T2Fs_intra_original_gldm_LargeDependenceHighGrayLevelEmphasis_intra
	ImageFusion 2mm	T2Fs_intra_wavelet_HHH_ngtdm_Strength
		T2Fs_intra_wavelet_LLL_gldm_LargeDependenceLowGrayLevelEmphasis
		T2Fs_intra_wavelet_LHH_ngtdm_Busyness
		T2Fs_intra_wavelet_HLH_gldm_LargeDependenceLowGrayLevelEmphasis
		T2Fs_intra_wavelet_LLL_glszm_LargeAreaLowGrayLevelEmphasis
		T2Fs_intra_wavelet_LLL_firstorder_TotalEnergy
		T2Fs_intra_wavelet_LHL_glszm_LargeAreaLowGrayLevelEmphasis
		T2Fs_intra_wavelet_LLH_ngtdm_Contrast
		T2Fs_intra_wavelet_HHL_gldm_LargeDependenceLowGrayLevelEmphasis
		T2Fs_intra_original_glszm_LargeAreaHighGrayLevelEmphasis
		T2Fs_intra_wavelet_LLL_ngtdm_Coarseness
		T2Fs_intra_original_gldm_LargeDependenceHighGrayLevelEmphasis
		T2Fs_intra_wavelet_LHL_ngtdm_Complexity
		T2Fs_intra_wavelet_LLL_glszm_SizeZoneNonUniformity
		T2Fs_intra_wavelet_HLH_firstorder_Mean
		T2Fs_intra_original_glszm_SmallAreaLowGrayLevelEmphasis
		T2Fs_intra_wavelet_LLL_glszm_GrayLevelVariance
		T2Fs_intra_wavelet_LHL_gldm_LargeDependenceLowGrayLevelEmphasis
		T2Fs_intra_wavelet_HLL_ngtdm_Complexity
		T2Fs_intra_original_glrlm_ShortRunLowGrayLevelEmphasis
		T2Fs_intra_wavelet_HLH_ngtdm_Strength
		T2Fs_intra_wavelet_LLH_gldm_LargeDependenceHighGrayLevelEmphasis
		T2Fs_intra_original_glszm_LowGrayLevelZoneEmphasis
		T2Fs_intra_wavelet_HLH_glszm_SmallAreaLowGrayLevelEmphasis
		T2Fs_intra_wavelet_LHL_glcm_Imc2
		T2Fs_intra_wavelet_HHH_glszm_SmallAreaHighGrayLevelEmphasis
		T2Fs_intra_wavelet_HHL_glcm_Idn
		T2Fs_intra_log_sigma_3_0_mm_3D_glcm_Correlation
T1CET2Fs	Intra	T1CE_intra_original_gldm_DependenceVariance
		T1CE_intra_wavelet_LLL_ngtdm_Coarseness
		T2Fs_intra_wavelet_LHH_ngtdm_Contrast
		T2Fs_intra_wavelet_LLL_firstorder_TotalEnergy
		T1CE_intra_wavelet_HLL_gldm_LargeDependenceHighGrayLevelEmphasis
		T1CE_intra_log_sigma_5_0_mm_3D_gldm_LargeDependenceLowGrayLevelEmphasis
		T2Fs_intra_wavelet_LLL_glszm_SizeZoneNonUniformity
		T2Fs_intra_wavelet_LLH_gldm_LargeDependenceLowGrayLevelEmphasis

	T2Fs_intra_original_gldm_LargeDependenceHighGrayLevelEmphasis
Peri2mm	T2Fs_peri_wavelet_LLL_glszm_LargeAreaHighGrayLevelEmphasis<br>T1CE_peri_original_firstorder_Skewness<br>T2Fs_peri_wavelet_LLL_gldm_LargeDependenceLowGrayLevelEmphasis<br>T2Fs_peri_original_shape_SurfaceVolumeRatio<br>T2Fs_peri_wavelet_HLH_glszm_LargeAreaLowGrayLevelEmphasis<br>T1CE_peri_wavelet_HLL_ngtdm_Contrast<br>T1CE_peri_log_sigma_5_0_mm_3D_glszm_LargeAreaHighGrayLevelEmphasis<br>T1CE_peri_wavelet_HLH_firstorder_Maximum<br>T1CE_peri_wavelet_LLH_firstorder_Kurtosis<br>T1CE_peri_wavelet_LHH_firstorder_Mean<br>T2Fs_peri_wavelet_LLL_firstorder_TotalEnergy<br>T1CE_peri_log_sigma_3_0_mm_3D_glcm_ClusterShade<br>T1CE_peri_wavelet_LLL_ngtdm_Coarseness<br>T1CE_peri_wavelet_HLL_glszm_LargeAreaLowGrayLevelEmphasis<br>T1CE_peri_wavelet_LLL_glszm_ZoneVariance
IntraPeri2mm	T1CE_intra_original_gldm_DependenceVariance<br>T1CE_peri_wavelet_HLL_ngtdm_Contrast<br>T1CE_peri_wavelet_LHH_glszm_LargeAreaHighGrayLevelEmphasis<br>T1CE_peri_log_sigma_3_0_mm_3D_glszm_ZoneVariance<br>T1CE_peri_log_sigma_3_0_mm_3D_glcm_ClusterShade<br>T2Fs_peri_wavelet_LLL_gldm_LargeDependenceLowGrayLevelEmphasis<br>T1CE_peri_wavelet_HLL_glszm_LargeAreaLowGrayLevelEmphasis<br>T1CE_peri_wavelet_LLL_firstorder_Skewness<br>T2Fs_peri_wavelet_HLH_glszm_LargeAreaLowGrayLevelEmphasis<br>T2Fs_intra_wavelet_LLH_ngtdm_Strength<br>T1CE_peri_wavelet_HLH_firstorder_Maximum<br>T2Fs_intra_wavelet_LLL_firstorder_TotalEnergy<br>T1CE_peri_wavelet_LHH_firstorder_Mean<br>T2Fs_peri_original_shape_SurfaceVolumeRatio<br>T1CE_peri_log_sigma_5_0_mm_3D_glszm_LargeAreaHighGrayLevelEmphasis<br>T2Fs_intra_wavelet_LHH_ngtdm_Contrast<br>T1CE_intra_wavelet_LHH_ngtdm_Busyness
ImageFusion 2mm	T1CE_intra_wavelet_HHH_firstorder_Median<br>T2Fs_intra_original_gldm_LargeDependenceHighGrayLevelEmphasis<br>T2Fs_intra_wavelet_LLL_ngtdm_Coarseness<br>T2Fs_intra_wavelet_LLL_firstorder_TotalEnergy<br>T1CE_intra_wavelet_HLL_gldm_LargeDependenceHighGrayLevelEmphasis

	T2Fs_intra_wavelet_HHL_gldm_LargeDependenceLowGrayLevelEmphasis
	T1CE_intra_log_sigma_5_0_mm_3D_gldm_LargeDependenceLowGrayLevelEmphasis
	T2Fs_intra_wavelet_LLH_gldm_LargeDependenceLowGrayLevelEmphasis
	T2Fs_intra_wavelet_LHL_ngtdm_Complexity
	T2Fs_intra_wavelet_LLL_glszm_SizeZoneNonUniformity
	T1CE_intra_wavelet_HLH_gldm_LargeDependenceHighGrayLevelEmphasis
	T1CE_intra_wavelet_HHH_glszm_SmallAreaLowGrayLevelEmphasis

Abbreviation: T1CE, T1-weighted contrast-enhanced; T2Fs, T2-weighted fat saturated; Intra, Intratumoral regions; Peri, Peritumoral regions; glszm, Gray Level Size Zone Matrix; ngtdm, Neighboring Gray Tone Difference Matrix; gldm, Gray Level Dependence Matrix; glcm, Gray Level Co-occurrence Matrix; glrlm, Gray Level Run Length Matrix; HLH, High-Low-High; HLL, High-Low-Low; LHH, Low-High-High; LLL, Low-Low-Low; HHH, High-High-High; HHL, High-High-Low; HLL, High-Low-Low; LHL, Low-High-Low; LLH, Low-Low-High.

Table S3 Univariate and multivariable logistic regression analyses for selecting clinical features of model development

Variable	Univariate analysis		Multivariate analysis	
	OR (95% CI)	p_value	OR (95% CI)	p_value
M	0.631(0.537, 0.739)	0.000	0.546(0.191, 1.565)	0.345
sex	0.685(0.597, 0.786)	0.000	0.935(0.604, 1.448)	0.799
size	0.713(0.634, 0.803)	0.000	0.562(0.341, 0.925)	0.057
fusion_gene	0.722(0.624, 0.836)	0.000	2.145(1.257, 3.662)	0.019
pathology	0.723(0.641, 0.816)	0.000	0.625(0.422, 0.924)	0.048
site	0.73(0.649, 0.82)	0.000	0.96(0.604, 1.527)	0.885
N	0.734(0.647, 0.833)	0.000	1.409(0.881, 2.255)	0.23
T	0.746(0.668, 0.833)	0.000	1.547(0.863, 2.773)	0.218
IRS	0.838(0.787, 0.891)	0.000	0.874(0.399, 1.916)	0.777
age	0.995(0.992, 0.997)	0.000	1.002(0.998, 1.007)	0.403

Abbreviation: IRS: Intergroup Rhabdomyosarcoma Study postsurgical grouping system; CI: confidence interval

Table S4 Performance of different T1CE models across the three datasets

Model	Accuracy	AUC	95% CI	Sensitivity	Specificity	PPV	NPV	Precision	Recall	F1	Threshold	Cohort
T1CE_Intra	0.869	0.94	0.9145 - 0.9647	0.896	0.853	0.779	0.935	0.779	0.896	0.833	0.381	train
T1CE_Peri2mm	0.838	0.926	0.8983 - 0.9546	0.906	0.799	0.722	0.936	0.722	0.906	0.803	0.373	train
T1CE_IntraPeri2mm	0.848	0.917	0.8848 - 0.9483	0.792	0.88	0.792	0.88	0.792	0.792	0.792	0.434	train
T1CE_ImageFusion2mm	0.831	0.927	0.8982 - 0.9553	0.896	0.793	0.714	0.93	0.714	0.896	0.795	0.385	train
T1CE_Intra	0.52	0.621	0.5175 - 0.7239	0.816	0.391	0.369	0.829	0.369	0.816	0.508	0.286	val
T1CE_Peri2mm	0.72	0.753	0.6606 - 0.8449	0.711	0.724	0.529	0.851	0.529	0.711	0.607	0.415	val
T1CE_IntraPeri2mm	0.672	0.76	0.6736 - 0.8463	0.816	0.609	0.477	0.883	0.477	0.816	0.602	0.389	val
T1CE_ImageFusion2mm	0.64	0.62	0.5098 - 0.7309	0.605	0.655	0.434	0.792	0.434	0.605	0.505	0.406	val
T1CE_Intra	0.689	0.559	0.3963 - 0.7226	0.182	0.974	0.8	0.679	0.8	0.182	0.296	0.681	test1
T1CE_Peri2mm	0.721	0.705	0.5555 - 0.8548	0.636	0.769	0.609	0.789	0.609	0.636	0.622	0.395	test1
T1CE_IntraPeri2mm	0.754	0.741	0.5973 - 0.8852	0.591	0.846	0.684	0.786	0.684	0.591	0.634	0.52	test1
T1CE_ImageFusion2mm	0.623	0.555	0.3950 - 0.7146	0.455	0.718	0.476	0.7	0.476	0.455	0.465	0.485	test1
T1CE_Intra	0.488	0.464	0.2905 - 0.6381	0.933	0.25	0.4	0.875	0.4	0.933	0.56	0.24	test2
T1CE_Peri2mm	0.721	0.752	0.5966 - 0.9082	0.533	0.821	0.615	0.767	0.615	0.533	0.571	0.425	test2
T1CE_IntraPeri2mm	0.791	0.843	0.7157 - 0.9700	0.667	0.857	0.714	0.828	0.714	0.667	0.69	0.445	test2
T1CE_ImageFusion2mm	0.465	0.498	0.3182 - 0.6770	0.8	0.286	0.375	0.727	0.375	0.8	0.511	0.265	test2

Abbreviation: T1CE, T1-weighted contrast-enhanced; Intra, Intratumoral regions; Peri, Peritumoral regions; AUC, Area under the receiver operator characteristic curve; CI, Confidence Interval; PPV, Positive Predictive Value; NPV, Negative Predictive Value; F1, F1 Score.

Table S5 Comparison of T1CE models using the Delong test, Net Reclassification Improvement and Integrated Discrimination Improvement across the three datasets

Model 1	Model 2	Delong p-value	NRI	NRI 95% CI	NRI p-value	IDI	IDI 95% CI	IDI p-value	Cohort
T1CE_Peri2mm	T1CE_Intra	0.021	0.171	(-0.192, 0.566)	0.375	0.184	(0.037, 0.33)	0.014	train
T1CE_IntraPeri2mm	T1CE_Intra	0	0.34	(-0.019, 0.674)	0.054	0.177	(0.082, 0.27)	0	train
T1CE_IntraPeri2mm	T1CE_Peri2mm	0.342	0.169	(-0.241, 0.582)	0.421	-0.007	(-0.118, 0.095)	0.893	train
T1CE_ImageFusion2mm	T1CE_Intra	0.774	-0.098	(-0.474, 0.267)	0.606	0.029	(-0.106, 0.172)	0.685	train
T1CE_ImageFusion2mm	T1CE_Peri2mm	0.007	-0.269	(-0.669, 0.115)	0.179	-0.155	(-0.264, -0.052)	0.004	train
T1CE_ImageFusion2mm	T1CE_IntraPeri2mm	0	-0.438	(-0.821, -0.024)	0.031	-0.148	(-0.248, -0.056)	0.003	train
T1CE_Peri2mm	T1CE_Intra	0.021	0.171	(-0.197, 0.562)	0.376	0.184	(0.028, 0.344)	0.022	val
T1CE_IntraPeri2mm	T1CE_Intra	0	0.34	(-0.002, 0.69)	0.054	0.177	(0.087, 0.264)	0	val
T1CE_IntraPeri2mm	T1CE_Peri2mm	0.342	0.169	(-0.199, 0.579)	0.394	-0.007	(-0.117, 0.102)	0.896	val
T1CE_ImageFusion2mm	T1CE_Intra	0.774	-0.098	(-0.44, 0.25)	0.579	0.029	(-0.114, 0.17)	0.691	val
T1CE_ImageFusion2mm	T1CE_Peri2mm	0.007	-0.269	(-0.656, 0.142)	0.186	-0.155	(-0.268, -0.049)	0.005	val
T1CE_ImageFusion2mm	T1CE_IntraPeri2mm	0	-0.438	(-0.817, -0.046)	0.026	-0.148	(-0.242, -0.053)	0.002	val
T1CE_Peri2mm	T1CE_Intra	0.021	0.171	(-0.192, 0.55)	0.365	0.184	(0.023, 0.342)	0.024	test1
T1CE_IntraPeri2mm	T1CE_Intra	0	0.34	(-0.022, 0.665)	0.052	0.177	(0.081, 0.274)	0	test1
T1CE_IntraPeri2mm	T1CE_Peri2mm	0.342	0.169	(-0.273, 0.569)	0.431	-0.007	(-0.115, 0.102)	0.895	test1
T1CE_ImageFusion2mm	T1CE_Intra	0.774	-0.098	(-0.435, 0.248)	0.575	0.029	(-0.111, 0.167)	0.685	test1
T1CE_ImageFusion2mm	T1CE_Peri2mm	0.007	-0.269	(-0.636, 0.118)	0.162	-0.155	(-0.263, -0.051)	0.004	test1
T1CE_ImageFusion2mm	T1CE_IntraPeri2mm	0	-0.438	(-0.817, -0.051)	0.025	-0.148	(-0.244, -0.051)	0.003	test1

T1CE_Peri2mm	T1CE_Intra	0.021	0.171	(-0.215, 0.578)	0.397	0.184	(0.02, 0.345)	0.026	test2
T1CE_IntraPeri2mm	T1CE_Intra	0	0.34	(-0.016, 0.664)	0.05	0.177	(0.089, 0.267)	0	test2
T1CE_IntraPeri2mm	T1CE_Peri2mm	0.342	0.169	(-0.244, 0.571)	0.416	-0.007	(-0.108, 0.108)	0.894	test2
T1CE_ImageFusion2mm	T1CE_Intra	0.774	-0.098	(-0.476, 0.25)	0.598	0.029	(-0.107, 0.165)	0.678	test2
T1CE_ImageFusion2mm	T1CE_Peri2mm	0.007	-0.269	(-0.677, 0.131)	0.192	-0.155	(-0.26, -0.047)	0.004	test2
T1CE_ImageFusion2mm	T1CE_IntraPeri2mm	0	-0.438	(-0.834, -0.069)	0.025	-0.148	(-0.242, -0.054)	0.002	test2

Abbreviation: T1CE, T1-weighted contrast-enhanced; Intra, Intratumoral regions; Peri, Peritumoral regions; NRI, Net reclassification improvement; CI, Confidence Interval; IDI, Integrated discrimination improvement.

Table S6 Performance of different T2Fs models across the three datasets

Model	Accuracy	AUC	95% CI	Sensitivity	Specificity	PPV	NPV	Precision	Recall	F1	Threshold	Cohort
T2Fs_Intra	0.828	0.886	0.8462 - 0.9251	0.698	0.902	0.804	0.838	0.804	0.698	0.747	0.442	train
T2Fs_Peri2mm	0.841	0.919	0.8888 - 0.9493	0.868	0.826	0.742	0.916	0.742	0.868	0.8	0.352	train
T2Fs_IntraPeri2mm	0.841	0.928	0.8985 - 0.9582	0.925	0.793	0.721	0.948	0.721	0.925	0.81	0.383	train
T2Fs_ImageFusion2mm	0.921	0.968	0.9491 - 0.9874	0.896	0.935	0.888	0.94	0.888	0.896	0.892	0.426	train
T2Fs_Intra	0.44	0.594	0.4875 - 0.7003	0.921	0.23	0.343	0.87	0.343	0.921	0.5	0.194	val
T2Fs_Peri2mm	0.624	0.559	0.4458 - 0.6725	0.316	0.759	0.364	0.717	0.364	0.316	0.338	0.424	val
T2Fs_IntraPeri2mm	0.6	0.597	0.4892 - 0.7056	0.553	0.621	0.389	0.761	0.389	0.553	0.457	0.352	val
T2Fs_ImageFusion2mm	0.704	0.598	0.4879 - 0.7075	0.263	0.897	0.526	0.736	0.526	0.263	0.351	0.451	val
T2Fs_Intra	0.492	0.592	0.4434 - 0.7408	0.909	0.256	0.408	0.833	0.408	0.909	0.563	0.223	test1
T2Fs_Peri2mm	0.607	0.614	0.4672 - 0.7601	0.545	0.641	0.462	0.714	0.462	0.545	0.5	0.321	test1
T2Fs_IntraPeri2mm	0.721	0.706	0.5616 - 0.8498	0.636	0.769	0.609	0.789	0.609	0.636	0.622	0.375	test1
T2Fs_ImageFusion2mm	0.689	0.721	0.5922 - 0.8507	0.636	0.718	0.56	0.778	0.56	0.636	0.596	0.355	test1
T2Fs_Intra	0.558	0.636	0.4580 - 0.8134	0.667	0.5	0.417	0.737	0.417	0.667	0.513	0.31	test2
T2Fs_Peri2mm	0.651	0.667	0.4859 - 0.8474	0.667	0.643	0.5	0.783	0.5	0.667	0.571	0.299	test2
T2Fs_IntraPeri2mm	0.744	0.76	0.6031 - 0.9159	0.667	0.786	0.625	0.815	0.625	0.667	0.645	0.436	test2
T2Fs_ImageFusion2mm	0.651	0.558	0.3719 - 0.7448	0.267	0.857	0.5	0.686	0.5	0.267	0.348	0.421	test2

Abbreviation: T2Fs, T2-weighted fat saturated; Intra, Intratumoral regions; Peri, Peritumoral regions; AUC, Area under the receiver operator characteristic curve; CI, Confidence Interval; PPV, Positive Predictive Value; NPV, Negative Predictive Value; F1, F1 Score.

Table S7 Comparison of T2Fs models using the Delong test, Net Reclassification Improvement and Integrated Discrimination Improvement across the three datasets

Model 1	Model 2	Delong p-value	NRI	NRI 95% CI	NRI p-value	IDI	IDI 95% CI	IDI p-value	Cohort
T2Fs_Peri2mm	T2Fs_Intra	0.797	0.143	(-0.271, 0.552)	0.496	0.039	(-0.102, 0.184)	0.593	train
T2Fs_IntraPeri2mm	T2Fs_Intra	0.311	0.286	(-0.109, 0.685)	0.158	0.085	(-0.042, 0.202)	0.172	train
T2Fs_IntraPeri2mm	T2Fs_Peri2mm	0.274	0.143	(-0.182, 0.464)	0.386	0.046	(-0.052, 0.153)	0.379	train
T2Fs_ImageFusion2mm	T2Fs_Intra	0.43	-0.043	(-0.396, 0.334)	0.818	-0.034	(-0.143, 0.061)	0.516	train
T2Fs_ImageFusion2mm	T2Fs_Peri2mm	0.262	-0.186	(-0.503, 0.117)	0.24	-0.073	(-0.173, 0.032)	0.164	train
T2Fs_ImageFusion2mm	T2Fs_IntraPeri2mm	0.04	-0.329	(-0.627, -0.039)	0.028	-0.119	(-0.204, -0.031)	0.007	train
T2Fs_Peri2mm	T2Fs_Intra	0.797	0.143	(-0.231, 0.552)	0.474	0.039	(-0.102, 0.176)	0.582	val
T2Fs_IntraPeri2mm	T2Fs_Intra	0.311	0.286	(-0.097, 0.685)	0.152	0.085	(-0.028, 0.205)	0.152	val
T2Fs_IntraPeri2mm	T2Fs_Peri2mm	0.274	0.143	(-0.195, 0.495)	0.417	0.046	(-0.051, 0.15)	0.37	val
T2Fs_ImageFusion2mm	T2Fs_Intra	0.43	-0.043	(-0.376, 0.316)	0.808	-0.034	(-0.121, 0.07)	0.488	val
T2Fs_ImageFusion2mm	T2Fs_Peri2mm	0.262	-0.186	(-0.493, 0.109)	0.227	-0.073	(-0.179, 0.028)	0.168	val
T2Fs_ImageFusion2mm	T2Fs_IntraPeri2mm	0.04	-0.329	(-0.631, 0.0)	0.041	-0.119	(-0.199, -0.033)	0.005	val
T2Fs_Peri2mm	T2Fs_Intra	0.797	0.143	(-0.241, 0.544)	0.476	0.039	(-0.104, 0.178)	0.587	test1
T2Fs_IntraPeri2mm	T2Fs_Intra	0.311	0.286	(-0.111, 0.687)	0.16	0.085	(-0.046, 0.204)	0.182	test1
T2Fs_IntraPeri2mm	T2Fs_Peri2mm	0.274	0.143	(-0.174, 0.484)	0.395	0.046	(-0.053, 0.162)	0.402	test1
T2Fs_ImageFusion2mm	T2Fs_Intra	0.43	-0.043	(-0.391, 0.317)	0.812	-0.034	(-0.13, 0.066)	0.499	test1
T2Fs_ImageFusion2mm	T2Fs_Peri2mm	0.262	-0.186	(-0.505, 0.124)	0.247	-0.073	(-0.177, 0.027)	0.161	test1
T2Fs_ImageFusion2mm	T2Fs_IntraPeri2mm	0.04	-0.329	(-0.639, -0.033)	0.034	-0.119	(-0.196, -0.035)	0.004	test1
T2Fs_Peri2mm	T2Fs_Intra	0.797	0.143	(-0.251, 0.574)	0.497	0.039	(-0.093, 0.186)	0.583	test2
T2Fs_IntraPeri2mm	T2Fs_Intra	0.311	0.286	(-0.079, 0.656)	0.128	0.085	(-0.037, 0.207)	0.172	test2
T2Fs_IntraPeri2mm	T2Fs_Peri2mm	0.274	0.143	(-0.2, 0.495)	0.42	0.046	(-0.054, 0.162)	0.404	test2
T2Fs_ImageFusion2mm	T2Fs_Intra	0.43	-0.043	(-0.383, 0.324)	0.812	-0.034	(-0.132, 0.06)	0.49	test2
T2Fs_ImageFusion2mm	T2Fs_Peri2mm	0.262	-0.186	(-0.5, 0.105)	0.229	-0.073	(-0.172, 0.027)	0.151	test2
T2Fs_ImageFusion2mm	T2Fs_IntraPeri2mm	0.04	-0.329	(-0.643, -0.039)	0.033	-0.119	(-0.198, -0.026)	0.007	test2

Abbreviation: T2Fs, T2-weighted fat saturated; Intra, Intratumoral regions; Peri, Peritumoral regions; NRI, Net reclassification improvement; CI, Confidence Interval; IDI, Integrated discrimination improvement.

Table S8 Performance of different T1CET2Fs models across the four datasets

Model	Accuracy	AUC	95% CI	Sensitivity	Specificity	PPV	NPV	Precision	Recall	F1	Threshold	Cohort
T1CET2Fs_Intra	0.879	0.939	0.9106 - 0.9681	0.915	0.859	0.789	0.946	0.789	0.915	0.847	0.415	train
T1CET2Fs_Peri2mm	0.879	0.942	0.9173 - 0.9673	0.792	0.929	0.866	0.886	0.866	0.792	0.828	0.443	train
T1CET2Fs_IntraPeri2mm	0.855	0.927	0.8965 - 0.9574	0.868	0.848	0.767	0.918	0.767	0.868	0.814	0.368	train
T1CET2Fs_ImageFusion2mm	0.824	0.926	0.8980 - 0.9546	0.934	0.761	0.692	0.952	0.692	0.934	0.795	0.377	train
T1CET2Fs_Intra	0.632	0.678	0.5733 - 0.7821	0.684	0.609	0.433	0.815	0.433	0.684	0.531	0.372	val
T1CET2Fs_Peri2mm	0.728	0.712	0.6055 - 0.8185	0.553	0.805	0.553	0.805	0.553	0.553	0.553	0.436	val
T1CET2Fs_IntraPeri2mm	0.848	0.855	0.7756 - 0.9340	0.684	0.92	0.788	0.87	0.788	0.684	0.732	0.508	val
T1CET2Fs_ImageFusion2mm	0.664	0.592	0.4798 - 0.7047	0.368	0.793	0.437	0.742	0.437	0.368	0.4	0.416	val
T1CET2Fs_Intra	0.639	0.517	0.3528 - 0.6810	0.364	0.795	0.5	0.689	0.5	0.364	0.421	0.512	test1
T1CET2Fs_Peri2mm	0.77	0.772	0.6421 - 0.9021	0.5	0.923	0.786	0.766	0.786	0.5	0.611	0.465	test1
T1CET2Fs_IntraPeri2mm	0.836	0.837	0.7222 - 0.9515	0.727	0.897	0.8	0.854	0.8	0.727	0.762	0.499	test1
T1CET2Fs_ImageFusion2mm	0.623	0.5	0.3447 - 0.6553	0	0.974	0	0.633	0	0		0.677	test1
T1CET2Fs_Intra	0.651	0.664	0.4923 - 0.8363	0.6	0.679	0.5	0.76	0.5	0.6	0.545	0.389	test2
T1CET2Fs_Peri2mm	0.721	0.808	0.6791 - 0.9376	0.867	0.643	0.565	0.9	0.565	0.867	0.684	0.397	test2
T1CET2Fs_IntraPeri2mm	0.86	0.838	0.6870 - 0.9892	0.667	0.964	0.909	0.844	0.909	0.667	0.769	0.508	test2
T1CET2Fs_ImageFusion2mm	0.465	0.504	0.3233 - 0.6838	0.867	0.25	0.382	0.778	0.382	0.867	0.531	0.179	test2

Abbreviation: T1CE, T1-weighted contrast-enhanced; T2Fs, T2-weighted fat saturated; Intra, Intratumoral regions; Peri, Peritumoral regions; AUC, Area under the receiver operator characteristic curve; CI, Confidence Interval; PPV, Positive Predictive Value; NPV, Negative Predictive Value; F1, F1 Score.

Table S9 Comparison of T1CET2Fs models using the Delong test, Net Reclassification Improvement, Integrated Discrimination Improvement and likelihood ratio test across the four datasets

Model 1	Model 2	Delong p-value	NRI	NRI 95% CI	NRI p-value	IDI	IDI 95% CI	IDI p-value	LRT	LRT 95% CI	LRT p-value	Cohort
T1CET2Fs_Peri2mm	T1CET2Fs_Intra	0.116	0.231	(-0.143, 0.603)	0.225	0.047	(-0.086, 0.174)	0.478	6.416	(-4.657, 18.244)	0.011	train
T1CET2Fs_IntraPeri2mm	T1CET2Fs_Intra	0.061	0.352	(-0.017, 0.736)	0.067	0.098	(-0.016, 0.217)	0.1	8.903	(-0.605, 21.134)	0.003	train
T1CET2Fs_IntraPeri2mm	T1CET2Fs_Peri2mm	0.675	0.121	(-0.141, 0.39)	0.37	0.051	(-0.045, 0.151)	0.31	2.487	(-3.838, 8.912)	0.115	train
T1CET2Fs_ImageFusion2mm	T1CET2Fs_Intra	0.019	-0.162	(-0.5, 0.195)	0.361	-0.103	(-0.182, -0.021)	0.012	-8.601	(-16.224, -1.31)	1	train
T1CET2Fs_ImageFusion2mm	T1CET2Fs_Peri2mm	0.003	-0.393	(-0.697, -0.069)	0.014	-0.15	(-0.282, -0.034)	0.018	-15.017	(-28.493, -2.546)	1	train
T1CET2Fs_ImageFusion2mm	T1CET2Fs_IntraPeri2mm	0.003	-0.514	(-0.823, -0.178)	0.002	-0.201	(-0.333, -0.082)	0.002	-17.505	(-32.99, -4.445)	1	train
T1CET2Fs_Peri2mm	T1CET2Fs_Intra	0.116	0.231	(-0.167, 0.602)	0.239	0.047	(-0.082, 0.175)	0.473	6.416	(-5.349, 20.045)	0.011	val
T1CET2Fs_IntraPeri2mm	T1CET2Fs_Intra	0.061	0.352	(-0.028, 0.733)	0.07	0.098	(-0.012, 0.229)	0.111	8.903	(-1.294, 21.066)	0.003	val
T1CET2Fs_IntraPeri2mm	T1CET2Fs_Peri2mm	0.675	0.121	(-0.16, 0.377)	0.375	0.051	(-0.042, 0.148)	0.295	2.487	(-4.088, 9.356)	0.115	val
T1CET2Fs_ImageFusion2mm	T1CET2Fs_Intra	0.019	-0.162	(-0.5, 0.195)	0.361	-0.103	(-0.184, -0.024)	0.011	-8.601	(-16.323, -1.037)	1	val
T1CET2Fs_ImageFusion2mm	T1CET2Fs_Peri2mm	0.003	-0.393	(-0.702, -0.055)	0.017	-0.15	(-0.272, -0.037)	0.012	-15.017	(-29.076, -2.032)	1	val
T1CET2Fs_ImageFusion2mm	T1CET2Fs_IntraPeri2mm	0.003	-0.514	(-0.822, -0.165)	0.002	-0.201	(-0.321, -0.084)	0.001	-17.505	(-30.946, -4.392)	1	val
T1CET2Fs_Peri2mm	T1CET2Fs_Intra	0.116	0.231	(-0.132, 0.601)	0.217	0.047	(-0.088, 0.168)	0.471	6.416	(-4.835, 18.596)	0.011	test1
T1CET2Fs_IntraPeri2mm	T1CET2Fs_Intra	0.061	0.352	(-0.044, 0.738)	0.077	0.098	(-0.012, 0.216)	0.092	8.903	(-1.455, 21.127)	0.003	test1
T1CET2Fs_IntraPeri2mm	T1CET2Fs_Peri2mm	0.675	0.121	(-0.151, 0.362)	0.353	0.051	(-0.049, 0.15)	0.317	2.487	(-4.254, 8.551)	0.115	test1
T1CET2Fs_ImageFusion2mm	T1CET2Fs_Intra	0.019	-0.162	(-0.511, 0.182)	0.36	-0.103	(-0.183, -0.023)	0.011	-8.601	(-16.085, -1.656)	1	test1
T1CET2Fs_ImageFusion2mm	T1CET2Fs_Peri2mm	0.003	-0.393	(-0.708, -0.091)	0.013	-0.15	(-0.278, -0.038)	0.014	-15.017	(-28.665, -3.176)	1	test1
T1CET2Fs_ImageFusion2mm	T1CET2Fs_IntraPeri2mm	0.003	-0.514	(-0.828, -0.163)	0.002	-0.201	(-0.323, -0.084)	0.001	-17.505	(-32.666, -4.647)	1	test1
T1CET2Fs_Peri2mm	T1CET2Fs_Intra	0.116	0.231	(-0.147, 0.595)	0.222	0.047	(-0.073, 0.178)	0.462	6.416	(-4.984, 20.399)	0.011	test2
T1CET2Fs_IntraPeri2mm	T1CET2Fs_Intra	0.061	0.352	(-0.038, 0.733)	0.073	0.098	(-0.016, 0.224)	0.11	8.903	(-1.779, 22.076)	0.003	test2
T1CET2Fs_IntraPeri2mm	T1CET2Fs_Peri2mm	0.675	0.121	(-0.141, 0.387)	0.367	0.051	(-0.038, 0.147)	0.282	2.487	(-3.614, 9.047)	0.115	test2
T1CET2Fs_ImageFusion2mm	T1CET2Fs_Intra	0.019	-0.162	(-0.505, 0.172)	0.349	-0.103	(-0.187, -0.016)	0.018	-8.601	(-15.952, -1.753)	1	test2
T1CET2Fs_ImageFusion2mm	T1CET2Fs_Peri2mm	0.003	-0.393	(-0.736, -0.059)	0.023	-0.15	(-0.271, -0.024)	0.017	-15.017	(-28.209, -1.657)	1	test2
T1CET2Fs_ImageFusion2mm	T1CET2Fs_IntraPeri2mm	0.003	-0.514	(-0.822, -0.149)	0.003	-0.201	(-0.319, -0.083)	0.001	-17.505	(-31.573, -4.852)	1	test2

Abbreviation: T1CE, T1-weighted contrast-enhanced; T2Fs, T2-weighted fat saturated; Intra, Intratumoral regions; Peri, Peritumoral regions; NRI, Net reclassification improvement; CI, Confidence Interval; IDI, Integrated discrimination improvement; LRT, Likelihood ratio test.

Table S10 Pathology subgroup analyses of T1CET2Fs_IntraPeri2mm model across the four datasets

Pathology	Accuracy	AUC	95% CI	Sensitivity	Specificity	PPV	NPV	Precision	Recall	F1	Threshold	Cohort
ERMS	0.872	0.925	0.925-0.925	0.830	0.896	0.815	0.905	0.815	0.830	0.822	0.420	train
ARMS	0.852	0.906	0.906-0.906	0.735	0.939	0.900	0.827	0.900	0.735	0.809	0.470	train
ScRMS	0.923	0.909	0.909-0.909	1.000	0.909	0.667	1.000	0.667	1.000	0.800	0.390	train
ERMS	0.730	0.801	0.801-0.801	0.867	0.688	0.464	0.943	0.464	0.867	0.605	0.420	val
ARMS	0.649	0.738	0.738-0.738	0.950	0.486	0.500	0.947	0.500	0.950	0.655	0.340	val
ScRMS	0.800	0.667	0.667-0.667	1.000	0.667	0.667	1.000	0.667	1.000	0.800	0.300	val
ERMS	0.800	0.852	0.852-0.852	0.750	0.833	0.750	0.833	0.750	0.750	0.750	0.450	test1
ARMS	0.846	0.812	0.812-0.812	0.857	0.842	0.667	0.941	0.667	0.857	0.750	0.510	test1
ScRMS	NA	NA	NA	NA	NA	NA	NA	NA	NA	NA	NA	test1
ERMS	0.769	0.804	0.804-0.804	0.889	0.706	0.615	0.923	0.615	0.889	0.727	0.400	test2
ARMS	0.692	0.767	0.767-0.767	1.000	0.600	0.429	1.000	0.429	1.000	0.600	0.310	test2
ScRMS	NA	NA	NA	NA	NA	NA	NA	NA	NA	NA	NA	test2

Abbreviation: ERMS, Embryonal rhabdomyosarcoma; ARMS, Alveolar rhabdomyosarcoma; ScRMS, Sclerosing/spindle cell rhabdomyosarcoma; AUC, Area under the receiver operator characteristic curve; CI, Confidence Interval; PPV, Positive Predictive Value; NPV, Negative Predictive Value.

Table S11 **Performance of the Clinic model, the T1CE_IntraPeri2mm model, and the combined model across the four datasets**

Model	Accuracy	AUC	95% CI	Sensitivity	Specificity	PPV	NPV	Cohort
Clinic	0.679	0.793	0.7400 - 0.8451	0.84	0.587	0.539	0.864	train
T1CE_IntraPeri2mm	0.848	0.917	0.8848 - 0.9483	0.792	0.88	0.792	0.88	train
Combined	0.845	0.927	0.8973 - 0.9565	0.868	0.832	0.748	0.916	train
Clinic	0.616	0.585	0.4769 - 0.6940	0.5	0.667	0.396	0.753	val
T1CE_IntraPeri2mm	0.672	0.76	0.6736 - 0.8463	0.816	0.609	0.477	0.883	val
Combined	0.664	0.746	0.6569 - 0.8358	0.816	0.598	0.47	0.881	val
Clinic	0.541	0.533	0.3809 - 0.6855	0.727	0.436	0.421	0.739	test1
T1CE_IntraPeri2mm	0.754	0.741	0.5973 - 0.8852	0.591	0.846	0.684	0.786	test1
Combined	0.738	0.753	0.6158 - 0.8900	0.591	0.821	0.65	0.78	test1
Clinic	0.698	0.621	0.4415 - 0.8014	0.4	0.857	0.6	0.727	test2
T1CE_IntraPeri2mm	0.791	0.843	0.7157 - 0.9700	0.667	0.857	0.714	0.828	test2
Combined	0.814	0.831	0.6959 - 0.9660	0.8	0.821	0.706	0.885	test2

Abbreviation: T1CE, T1-weighted contrast-enhanced; Intra, Intratumoral regions; Peri, Peritumoral regions; AUC, Area under the receiver operator characteristic curve; CI, Confidence Interval; PPV, Positive Predictive Value; NPV, Negative Predictive Value.

Table S12 **Comparison of the Clinic model, the T1CE_IntraPeri2mm model, and the combined model using the Delong test, Net Reclassification Improvement, Integrated Discrimination Improvement and likelihood ratio test across the three datasets**

Model 1	Model 2	Delong p-value	NRI	NRI 95% CI	NRI p-value	IDI	IDI 95% CI	IDI p-value	Cohort
T1CE_IntraPeri2mm	Clinic	0.066	0.267	(-0.143, 0.658)	0.192	0.122	(0.009, 0.243)	0.041	train
Combined	Clinic	0.081	0.364	(-0.023, 0.712)	0.052	0.366	(0.161, 0.562)	0	train
Combined	T1CE_IntraPeri2mm	0.553	0.098	(-0.067, 0.313)	0.314	0.245	(0.103, 0.361)	0	train
T1CE_IntraPeri2mm	Clinic	0.066	0.267	(-0.162, 0.672)	0.21	0.122	(0.003, 0.258)	0.061	val
Combined	Clinic	0.081	0.364	(-0.023, 0.739)	0.061	0.366	(0.149, 0.574)	0.001	val
Combined	T1CE_IntraPeri2mm	0.553	0.098	(-0.074, 0.313)	0.323	0.245	(0.105, 0.368)	0	val
T1CE_IntraPeri2mm	Clinic	0.066	0.267	(-0.164, 0.639)	0.193	0.122	(0.003, 0.236)	0.04	test1
Combined	Clinic	0.081	0.364	(-0.027, 0.726)	0.058	0.366	(0.145, 0.576)	0.001	test1
Combined	T1CE_IntraPeri2mm	0.553	0.098	(-0.069, 0.294)	0.292	0.245	(0.12, 0.362)	0	test1
T1CE_IntraPeri2mm	Clinic	0.066	0.267	(-0.18, 0.649)	0.207	0.122	(0.005, 0.25)	0.051	test2
Combined	Clinic	0.081	0.364	(-0.032, 0.727)	0.06	0.366	(0.154, 0.569)	0.001	test2
Combined	T1CE_IntraPeri2mm	0.553	0.098	(-0.062, 0.294)	0.282	0.245	(0.117, 0.361)	0	test2

Abbreviation: T1CE, T1-weighted contrast-enhanced; Intra, Intratumoral regions; Peri, Peritumoral regions; NRI, Net reclassification improvement; CI, Confidence Interval; IDI, Integrated discrimination improvement.

Appendix1: Radiomics Quality Score (RQS) 2.0 with Radiomics Readiness Levels (RRLs)

RRL 1 – Foundational Exploration

No.	Criteria	Options
1	Unmet Clinical Need – Unmet clinical need (UCN) defined.<br>● UCN is agreed upon and defined by more than one center.<br>● UCN is defined using an established consensus method such as the Delphi method.	○ Not implemented<br>◉ Implemented: More than one center (+1)<br>○ Implemented: Delphi method (+2)
2	Hardware Description – Detailed description of the imaging hardware used, including model, manufacturer, and technical specifications.	○ Not implemented<br>◉ Implemented (+1)
3	Image Protocol Quality – Five levels of image protocol quality for TRIAC:<br>● Level 0: Protocol not formally approved.<br>● Level 1: Approved with a reference number in the institutional archive.<br>● Level 2: Approved with formal quality assurance (recommended minimum for prospective trials).<br>● Level 3: Established internationally; published in guidelines and peer-reviewed papers.<br>● Level 4: Future proof (follows TRIAC Level 3, FAIR principles, retains raw data).	○ Not implemented<br>◉ Level 1/2 (+1)<br>○ Level 3/4 (+2)
4	Inclusion and Exclusion Criteria – Detailed criteria for patient selection in studies, including rationale.	○ Not implemented<br>◉ Implemented (+1)
5	Diversity and Distribution – Identify potential biases before the project (demographics, socioeconomic, geographic, medical profiles).	◉ Not implemented<br>○ Implemented (+1)

RRL 2 – Data Preparation

No.	Criteria	Options
6	Feature Robustness – Assess robustness via:<br>1. Imaging at multiple time points (test–retest).<br>2. Multiple segmentations (different physicians/algorithms/noise/perturbations).<br>3. Phantom study (identify inter-scanner/vendor differences).	○ Not implemented<br>◉ Implemented (+1)
7	Preprocessing of Images – Apply steps to standardize images with clear reasoning.	○ Not implemented<br>◉ Implemented (+1)
8	Harmonization – Use image-level (e.g. CycleGANs) or feature-level (e.g. ComBat) harmonization techniques.	◉ Not implemented<br>○ Implemented (+1)
9	Compliance with International Standards – Use implementations that adhere to standards (e.g., IBSI) for radiomic feature extraction.	○ Not implemented<br>◉ Implemented (+1)
10	Automatic Segmentation – Use an automated segmentation algorithm for ROI definition.	○ Not implemented<br>◉ Implemented (+1)

RRL 3 – Prototype Model Development

No.	Criteria	Options
11	Feature Reduction – Reduce features to lower the risk of overfitting (especially when features outnumber samples; check for correlations with volume).	○ Not implemented<br>◉ Implemented (+1)
12	Feature Robustness for Feature Selection – Integrate robustness evaluation into feature selection using prior test–retest, phantom, or segmentation studies.	○ Not implemented<br>◉ Implemented (+1)
13	HCR + DL Combination – Compare and explore the synergistic combination of handcrafted radiomics and deep learning models.	◉ Not implemented<br>○ Implemented (+1)
14	Multivariable Analysis – Incorporate non-radiomics features (clinical, genomic, proteomic) to yield a holistic model.	○ Not implemented<br>◉ Implemented (+2)

RRL 4 – Internal Validation

No.	Criteria	Options
15	Single Center Validation – Validation performed on data from the same institute without retraining or adapting the cut-off value.	○ Not implemented<br>◉ Implemented (+1)
16	Cut-off Analyses – Identify optimal thresholds (e.g., using Youden's Index) for classification or survival analysis.	◉ Not implemented<br>○ Implemented (+1)
17	Discrimination Statistics – Report discrimination metrics (e.g., ROC curve, sensitivity, specificity) with significance (p-values, CIs).<br>● Statistic reported<br>● With Resampling method	○ Not implemented<br>○ Statistic only (+1)<br>◉ Resampling method applied (+2)
18	Calibration Statistics – Report calibration metrics (e.g., calibration-in-the-large, slope, plots).	○ Not implemented<br>◉ Implemented (+1)
19	Failure Mode Analysis – Document model limitations with examples of edge cases.	◉ Not implemented<br>○ Implemented (+1)
20	Open Science and Data – Make code and data publicly available.<br>● Open scans (+1)<br>● Open segmentations (+1)<br>● Open code (+1)	○ Not implemented<br>◉ One aspect (+1)<br>○ Two aspects (+2)<br>○ All aspects (+3)

RRL 5 – Capability Testing

No.	Criteria	Options
21	Multi-center Validation – Validation with data from multiple institutes ensuring no overlap:<br>● One external institute<br>● Two or more external institutes<br>● Third-party platform with completely unseen data	○ Not implemented<br>○ One institute (+1)<br>◉ Two or more institutes (+2)<br>○ Third-party platform(+3)
22	Comparison with 'Current Clinical Standard' – Assess model agreement or superiority versus the current gold standard (e.g., TNM staging).	◉ Not implemented<br>○ Implemented (+2)
23	Comparison to Previous Work – Compare performance with published HCR signatures or DL algorithms.	◉ Not implemented<br>○ Implemented (+1)
24	Potential Clinical Utility – Report on the current and potential clinical application (e.g., decision curve analysis).	○ Not implemented<br>◉ Implemented (+2)

RRL 6 – Trustworthiness Assessment

No.	Criteria	Options
25	Explainability – Apply explainability tools (e.g., SHAP for HCR, GradCAM for DL) to clarify model predictions.	○ Not implemented<br>◉ Implemented (+1)
26	Explainability Evaluation – Conduct qualitative and quantitative evaluations of interpretability methods (e.g., checking consistency to adversarial perturbations).	◉ Not implemented<br>○ Implemented (+1)
27	Biological Correlates – Detect and discuss biological correlates to deepen understanding of radiomics and underlying biology.	○ Not implemented<br>◉ Implemented (+1)
28	Fairness Evaluation and Mitigation – Evaluate model performance for biases and apply bias correction if needed.<br>● Fairness evaluated<br>● Bias correction applied	◉ Not implemented<br>○ Fairness evaluated (+1)<br>○ With Bias correction (+2)

RRL 7 – Prospective Validity

No.	Criteria	Options
29	Usability for Clinicians – Evaluate the tool's usability, interface, and workflow integration.	◉ Not implemented<br>○ Implemented (+1)
30	Sample Size Calculation – Ensure statistical validity by calculating the appropriate sample size before prospective validation.	○ Not implemented<br>◉ Implemented (+1)
31	Clinical Trial Pre-registration – Register the prospective trial (including its statistical plan) on a clinical trial database (e.g., ClinicalTrials.gov).	◉ Not implemented<br>○ Implemented (+1)
32	Prospective Validation – Carry out prospective (or in silico) validation to ensure clinical validity of the biomarker.	◉ Not implemented<br>○ Implemented (+3)
33	Real-World Clinical Assessment – Conduct human-in-the-loop assessments to evaluate the practical impact of the radiomics model.	◉ Not implemented<br>○ Implemented (+1)

RRL 8 – Applicability and Sustainability

No.	Criteria	Options
34	Software Traceability – Implement and document a robust traceability process detailing development, changes, and version control.	◉ Not implemented ○ Implemented (+1)
35	Software Safeguards – Implement checks to prevent out-of-scope use or unreliable inputs.	◉ Not implemented ○ Implemented (+1)
36	Cost-effectiveness Analysis – Report on the cost-effectiveness of the clinical application (e.g., QALYs generated).	◉ Not implemented ○ Implemented (+2)
37	Performance Drift – Define a strategy to evaluate model performance periodically due to data shifts.	◉ Not implemented ○ Implemented (+1)
38	Continuous Learning – Define a strategy for continuous learning and improvement over time.	◉ Not implemented ○ Implemented (+1)

RRL 9 – Clinical Deployment

No.	Criteria	Options
39	Define the Level of Automation in Clinical Practice –<br>● Level 0: No Automation (clinician performs the task).<br>● Level 1: Clinical Assistance (model prediction assists).<br>● Level 2: Partial Automation (model prediction considered before final recommendation).<br>● Level 3: Conditional Automation (model provides predictions under supervision).<br>● Level 4: High Automation (predictions provided; clinician intervenes in special cases).<br>● Level 5: Full Automation (predictions provided without human intervention).	◉ Not implemented ○ Implemented (+1)
40	Quality Management System – Implement and maintain a QMS (e.g., ISO 9001) to ensure consistent quality and compliance.	◉ Not implemented ○ Implemented (+1)
41	Regulatory Requirements – Evaluate alignment with the chosen regulatory pathway (e.g., FDA 510(k), PMA, EMA, European AI Act).	◉ Not implemented ○ Implemented (+1)
42	Product on the Market – Successfully introduce the radiomics product to the market ensuring regulatory approval and clinical adoption.	◉ Not implemented ○ Implemented (+1)

Total Score: 24 out of 56 [43%]

Your AI tool is RRL 9 Compliant with a percentage of 43%

Hide Detailed Results

Evolution of Score by RRL Level

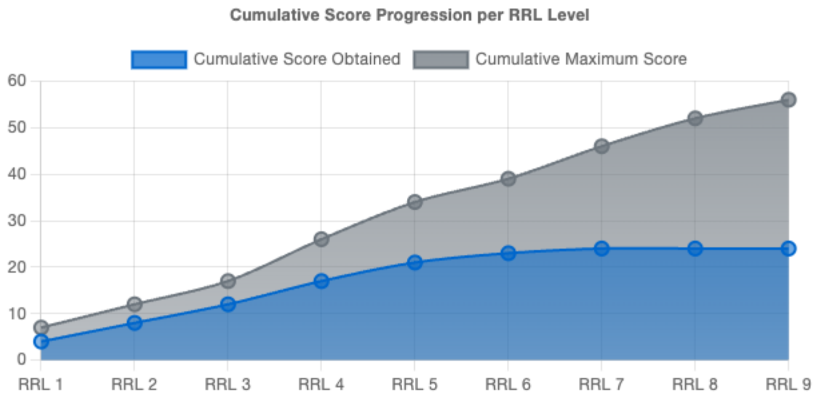

Points Satisfied per Stage

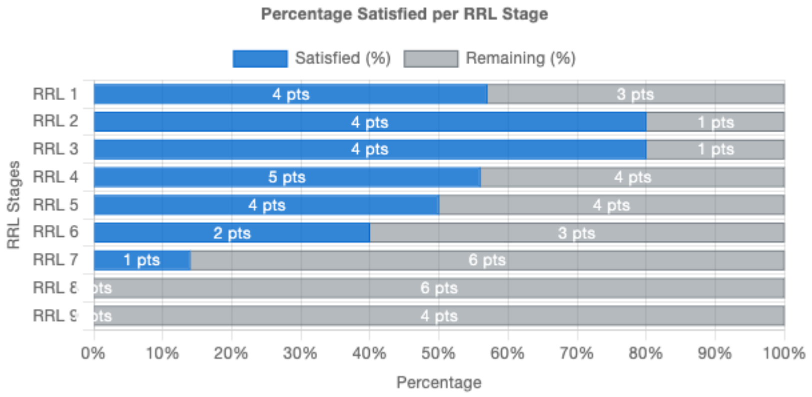

Appendix2: CheckList for EvaluAtion of Radiomics research (CLEAR)

CLEAR Checklist v1.0

Note: Use the checklist in conjunction with the main text for clarification of all items.
Yes, details provided; No, details not provided; n/e, not essential; n/a, not applicable; Page, page number

Section	No.	Item	Yes	No	n/a	Page
**Title**						
	1	Relevant title, specifying the radiomic methodology	☑	☐	☐	1
**Abstract**						
	2	Structured summary with relevant information	☑	☐	☐	1
**Keywords**						
	3	Relevant keywords for radiomics	☑	☐	☐	1
**Introduction**						
	4	Scientific or clinical background	☑	☐	☐	3
	5	Rationale for using a radiomic approach	☑	☐	☐	3-4
	6	Study objective(s)	☑	☐	☐	4
**Method**						
Study design	7	Adherence to guidelines or checklists (e.g., CLEAR checklist)	☑	☐	☐	Supp
	8	Ethical details (e.g., approval, consent, data protection)	☑	☐	☐	4
	9	Sample size calculation	☑	☐	☐	Supp
	10	Study nature (e.g., retrospective, prospective)	☑	☐	☐	4
	11	Eligibility criteria	☑	☐	☐	Supp
	12	Flowchart for technical pipeline	☑	☐	☐	Figur
Data	13	Data source (e.g., private, public)	☑	☐	☐	4
	14	Data overlap	☐	☑	☐	
	15	Data split methodology	☑	☐	☐	4
	16	Imaging protocol (i.e., image acquisition and processing)	☑	☐	☐	4
	17	Definition of non-radiomic predictor variables	☑	☐	☐	Supp
	18	Definition of the reference standard (i.e., outcome variable)	☑	☐	☐	Supp
Segmentation	19	Segmentation strategy	☑	☐	☐	Supp
	20	Details of operators performing segmentation	☑	☐	☐	Supp
Pre-processing	21	Image pre-processing details	☑	☐	☐	4
	22	Resampling method and its parameters	☑	☐	☐	4
	23	Discretization method and its parameters	☐	☐	☑	

Section	No.	Item	Yes	No	n/a	Page
	24	Image types (e.g., original, filtered, transformed)	☑	☐	☐	4
Feature extraction	25	Feature extraction method	☑	☐	☐	5
	26	Feature classes	☑	☐	☐	Supp
	27	Number of features	☑	☐	☐	Supp
	28	Default configuration statement for remaining parameters	☐	☑	☐	
Data preparation	29	Handling of missing data	☐	☐	☑	
	30	Details of class imbalance	☐	☐	☑	
	31	Details of segmentation reliability analysis	☑	☐	☐	Supp
	32	Feature scaling details (e.g., normalization, standardization)	☑	☐	☐	5
	33	Dimension reduction details	☑	☐	☐	5
Modeling	34	Algorithm details	☑	☐	☐	5
	35	Training and tuning details	☑	☐	☐	5
	36	Handling of confounders	☑	☐	☐	5
	37	Model selection strategy	☑	☐	☐	5
Evaluation	38	Testing technique (e.g., internal, external)	☑	☐	☐	5
	39	Performance metrics and rationale for choosing	☑	☐	☐	5
	40	Uncertainty evaluation and measures (e.g., confidence intervals)	☑	☐	☐	6
	41	Statistical performance comparison (e.g., DeLong's test)	☑	☐	☐	6
	42	Comparison with non-radiomic and combined methods	☑	☐	☐	5-6
	43	Interpretability and explainability methods	☑	☐	☐	6
**Results**						
	44	Baseline demographic and clinical characteristics	☑	☐	☐	6
	45	Flowchart for eligibility criteria	☑	☐	☐	Supp
	46	Feature statistics (e.g., reproducibility, feature selection)	☑	☐	☐	Supp
	47	Model performance evaluation	☑	☐	☐	6-8
	48	Comparison with non-radiomic and combined approaches	☑	☐	☐	8
**Discussion**						
	49	Overview of important findings	☑	☐	☐	9-10
	50	Previous works with differences from the current study	☑	☐	☐	10
	51	Practical implications	☑	☐	☐	12
	52	Strengths and limitations (e.g., bias and generalizability issues)	☑	☐	☐	11-12

Section	No.	Item	Yes	No	n/a	Page
**Open Science**						
Data availability	53	Sharing images along with segmentation data [n/e]	☐	☑	☐	
	54	Sharing radiomic feature data	☑	☐	☐	Supp
Code availability	55	Sharing pre-processing scripts or settings	☐	☑	☐	
	56	Sharing source code for modeling	☐	☑	☐	
Model availability	57	Sharing final model files	☐	☑	☐	
	58	Sharing a ready-to-use system [n/e]	☐	☑	☐	

Kocak B, Baessler B, Bakas S, Cuocolo R, Fedorov A, Maier-Hein L, Mercaldo N, Müller H, Orlhac F, Pinto Dos Santos D, Stanzione A, Ugga L, Zwanenburg A. CheckList for EvaluAtion of Radiomics research (CLEAR): a step-by-step reporting guideline for authors and reviewers endorsed by ESR and EuSoMII. Insights Imaging. 2023 May 4;14(1):75. doi: 10.1186/s13244-023-01415-8

Appendix3: METhodological RadiomICs Score (METRICS)

METRICS Tool v1.0

Please fill out all conditions first for relevant sections and then all active items to calculate METRICS score.

Please note that default option is "No".

? Stands for explanation of items and conditions.

C Stands for conditional items or sections.

Items/Conditions	Definitions	Weights	Options
**Study Design**			
Item#1	? Adherence to radiomics and/or machine learning-specific checklists or guidelines	0.0368	◉ Yes ○ No
Item#2	? Eligibility criteria that describe a representative study population	0.0735	◉ Yes ○ No
Item#3	? High-quality reference standard with a clear definition	0.0919	◉ Yes ○ No
**Imaging Data**			
Item#4	? Multi-center	0.0438	◉ Yes ○ No
Item#5	? Clinical translatability of the imaging data source for radiomics analysis	0.0292	◉ Yes ○ No
Item#6	? Imaging protocol with acquisition parameters	0.0438	◉ Yes ○ No
Item#7	? The interval between imaging used and reference standard	0.0292	○ Yes ◉ No
**Segmentation** C			
Condition#1	? Does the study include segmentation?		◉ Yes ○ No
Condition#2	? Does the study include fully automated segmentation?		○ Yes ◉ No
Item#8	? Transparent description of segmentation methodology	0.0337	◉ Yes ○ No
Item#9	? Formal evaluation of fully automated segmentation C	0.0225	○ Yes ○ No
Item#10	? Test set segmentation masks produced by a single reader or automated tool	0.0112	○ Yes ◉ No
**Image Processing and Feature Extraction**			
Condition#3	? Does the study include hand-crafted feature extraction?		◉ Yes ○ No
Item#11	? Appropriate use of image preprocessing techniques with transparent description	0.0622	◉ Yes ○ No
Item#12	? Use of standardized feature extraction software C	0.0311	◉ Yes ○ No
Item#13	? Transparent reporting of feature extraction parameters, otherwise providing a default configuration statement	0.0415	○ Yes ◉ No
**Feature Processing**			
Condition#4	? Does the study include tabular data?		◉ Yes ○ No
Condition#5	? Does the study include end-to-end deep learning?		○ Yes ◉ No
Item#14	? Removal of non-robust features C	0.0200	◉ Yes ○ No
Item#15	? Removal of redundant features C	0.0200	◉ Yes ○ No
Item#16	? Appropriateness of dimensionality compared to data size C	0.0300	◉ Yes ○ No
Item#17	? Robustness assessment of end-to-end deep learning pipelines C	0.0200	○ Yes ○ No
**Preparation for Modeling**			
Item#18	? Proper data partitioning process	0.0599	◉ Yes ○ No
Item#19	? Handling of confounding factors	0.0300	◉ Yes ○ No
**Metrics and Comparison**			
Item#20	? Use of appropriate performance evaluation metrics for task	0.0352	◉ Yes ○ No
Item#21	? Consideration of uncertainty	0.0234	◉ Yes ○ No
Item#22	? Calibration assessment	0.0176	◉ Yes ○ No
Item#23	? Use of uni-parametric imaging or proof of its inferiority	0.0117	◉ Yes ○ No
Item#24	? Comparison with a non-radiomic approach or proof of added clinical value	0.0293	◉ Yes ○ No
Item#25	? Comparison with simple or classical statistical models	0.0176	◉ Yes ○ No
**Testing**			
Item#26	? Internal testing	0.0375	◉ Yes ○ No
Item#27	? External testing	0.0749	◉ Yes ○ No
**Open Science**			
Item#28	? Data availability	0.0075	◉ Yes ○ No
Item#29	? Code availability	0.0075	○ Yes ◉ No
Item#30	? Model availability	0.0075	○ Yes ◉ No
		**Total METRICS score:**	**89.9%**
		? **Quality category:**	**Excellent**
		? **Publication ID:**	

If you publish any work which uses this tool, please cite the following publication:

Kocak B, Akinci D'Antonoli T, Mercaldo N, et al. METhodological RadiomICs Score (METRICS): a quality scoring tool for radiomics research endorsed by EuSoMII. Insights Imaging. 2024;15(1):8. Published 2024 Jan 17. doi:10.1186/s13244-023-01572-w
